# Optimizing microRNA delivery via albumin-decorated nanostructured lipid carriers

**DOI:** 10.1016/j.ijpx.2025.100441

**Published:** 2025-11-07

**Authors:** Ivana Ruseska, Amina Tucak-Smajić, Ivan Vidaković, Karin Kornmüller, Edina Vranić, Andreas Zimmer

**Affiliations:** a Department of Pharmaceutical technology and Biopharmacy, Institute of Pharmaceutical Sciences, University of Graz, Universitätsplatz 1, 8010 Graz, Austria; b Department of Pharmaceutical Technology, University of Sarajevo – Faculty of Pharmacy, Zmaja od Bosne 8, 71000, Bosnia and Herzegovina; c Division of Medical Physics and Biophysics, Gottfried Schatz Research Center for Cell Signaling, Metabolism and Aging, Medical University of Graz, Neue Stiftingtalstrasse 6, 8010 Graz, Austria

**Keywords:** Lipid nanoparticle, miRNA, Human serum albumin, Cellular uptake, Protein corona

## Abstract

microRNA-27a is a promising candidate for miRNA mimic therapy to combat obesity, but its clinical application is hindered by enzymatic degradation and low membrane permeability. To address these challenges, we developed cationic nanostructured lipid carriers (cNLCs) via high-pressure homogenization as non-viral carriers for miRNA-27a. However, the formation of a protein corona in biologically-relevant media altered the particle size and surface charge, significantly reducing cellular uptake. To mitigate this issue, we hypothesized that coating miRNA/cNLC complexes with human serum albumin (HSA) will prevent protein corona formation and enhance cellular uptake. The HSA-coated miRNA/cNLC complexes, termed albuplexes, were characterized for particle size, zeta potential, morphology, and stability in various media. The integrity of the HSA coat was assessed using circular dichroism and UV/Vis spectroscopy. We also evaluated the biocompatibility and cellular uptake of albuplexes in 3T3-L1 cells. The biological effects of miRNA-27a on adipocyte development were analyzed through light microscopy and absorbance measurements of Oil-red-O dye in lipid droplets. Results indicated that albuplexes possess favourable physicochemical properties and enhanced stability in serum. Notably, albuplexes were rapidly taken up by 3T3-L cells via endocytosis, although 20 % HSA in the culture medium completely inhibited uptake. Furthermore, albuplexes exhibited an anti-adipogenic effect by reducing the lipid droplet accumulation, suggesting their potential as a therapeutic strategy for miRNA replacement in obesity treatment.

## Highlights

• cNLCs were developed as carriers for anti-adipogenic miRNA-27a.
• HSA coating formed stable albuplexes and prevented protein corona formation.
• Albuplexes showed high stability and favourable properties in serum-containing media.
• Rapid cellular uptake of albuplexes occurred via endocytosis in 3T3-L1 cells.
• Albuplexes reduced lipid droplets accumulation during adipocyte differentiation.

## Graphical abstract

**Figure:**
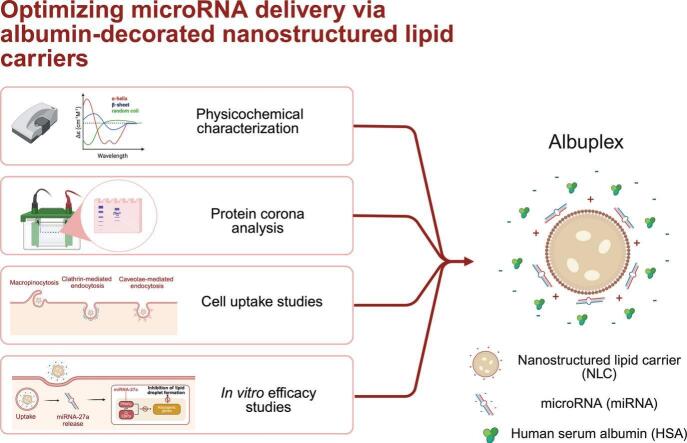

## 1 Introduction

Nucleic acid-based therapeutics represent a class of biopharmaceuticals that use RNA or DNA sequences to modulate gene expression and induce therapeutic effects (Kulkarni et al., 2021). To date, various molecules that downregulate gene expression, which include small interfering RNA (siRNA), antisense oligonucleotides (ASOs), short hairpin RNA (shRNA), and microRNA (miRNA)-based therapies, have been approved by the United States Food and Drug Administration (FDA) and the European Medicines Agency (EMA) or are in late-stage development for treating various diseases (Kulkarni et al., 2021; Mendes et al., 2022). The groundbreaking success of nucleic acid therapeutics has been demonstrated by two mRNA-based vaccines, BNT162b2 (Pfizer-BioNTech) and mRNA-1273 (Moderna), which use lipid nanoparticles (LNs) as delivery agents of messenger RNAs (mRNA)-encoding viral antigens, (Mendes et al., 2022; Wilson and Mukundan Geetha, 2022) as well as Onpattro®, the FDA-approved siRNA-based therapeutic for hereditary transthyretin-mediated amyloidosis (Hamilton et al., 2023).

Among nucleic acid therapeutics, miRNAs have gained significant attention due to their ability to regulate protein-encoding genes post-transcriptionally (Diener et al., 2022). miRNAs represent a class of small, non-coding endogenous RNA molecules, typically 20–24 nucleotides in length, that act by binding to target mRNAs, which lead to either translational repression or degradation of mRNA (Lee et al., 2019). It has been found that dysregulation of miRNA levels is associated with the onset and progress of various genetic, metabolic, and immunological diseases (Lee et al., 2019). Therefore, to modify miRNA levels, two therapeutic approaches have been developed—miRNA replacement therapy that utilizes synthetic miRNAs (miRNA mimics) and miRNA inhibition therapy that uses oligonucleotide-based miRNA inhibitors (anti-miRNAs) or recombinant expression vectors that carry miRNA-encoding sequences (Diener et al., 2022). While several siRNA-based therapeutics have already been approved or are in phase III clinical trials (Ahn et al., 2023; Traber and Yu, 2023), most miRNA-based therapies remain in preclinical or early clinical trial phases. Only a few candidates, including MRX34, a liposomal formulation of miR-34a mimic (NCT01829971; NCT02862145) and miravirsen (SPC3649), an antagomir targeting miR-122 (NCT01727934; NCT01872936; NCT01200420), have progressed to phase I or II trials (Diener et al., 2022; Traber and Yu, 2023).

**Fig. 1:**
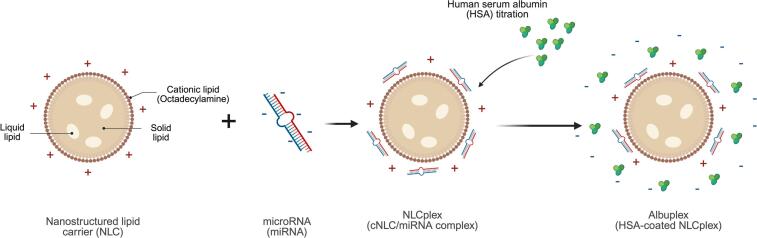
Schematic representation of albuplex formation through electrostatic complexation of cationic nanostructured lipid carriers (cNLCs) with microRNA (miRNA) to form NLCplexes. This is followed by surface decoration with human serum albumin (HSA) to obtain albuplexes.

Despite their potential, miRNA therapeutics face significant challenges, including poor cellular permeability, rapid degradation by serum endonucleases, and renal clearance due to their hydrophilic and anionic nature (Diener et al., 2022; Hou et al., 2021). To overcome these obstacles, various strategies have been explored, including chemical modifications, conjugation with biomolecules, as well as the development of efficient delivery systems (Diener et al., 2022). Delivery approaches are classified as viral-based carriers and non-viral carriers, such as peptide-based (Ruseska and Zimmer, 2023; Schachner-Nedherer et al., 2019) and lipid-based delivery systems (Ruseska et al., 2025; Tucak-Smajić et al., 2023). Nanostructured lipid carriers (NLCs) represent a second-generation lipid nanoparticle system, which is composed of a lipid matrix containing a mixture of solid and a liquid lipid, stabilized by surfactants. LNs can serve as miRNA carriers in two ways: (1) by encapsulating nucleic acids within the lipid matrix, as seen in COVID-19 vaccines, or (2) by adsorbing nucleic acids onto the surface of nanoparticles. Given that miRNAs are negatively charged molecules, our research group developed cationic NLCs (cNLC) as carriers for miRNA-27a, which formed miRNA/cNLC complexes through electrostatic interactions between miRNAs and the cationic lipid octadecylamine (OA), as presented in Fig. 1.(Ruseska et al., 2025; Tucak-Smajić et al., 2023). Our previous studies successfully demonstrated the delivery of antiadipogenic miRNA-27a to 3T3-L1 preadipocytes, effectively inhibiting their differentiation to mature adipocytes. In general, the advantages of our cNLCs include the use of fully GRAS components, the absence of organic solvents, and the ability to fine-tune surface charge and lipid composition for efficient and targeted miRNA complexation (Tucak-Smajić et al., 2023).

It is worth noting that the successful delivery of nucleic acids using non-viral carriers is limited by several rate-limiting steps, that include the interaction of complexes with the cellular membrane, internalization of complexes, and finally the release of cargo into the cytoplasm (Ponti et al., 2021). To exert their therapeutic effect, nucleic acids must successfully escape from endosomes and avoid lysosomal degradation (Hamilton et al., 2023). Therefore, when miRNAs are encapsulated within the lipid matrix of lipid nanoparticles, the particles must disassemble (e.g., LNs) or undergo structural reorganization (e.g., liposomes) to release their cargo. On the other hand, when miRNAs are adsorbed onto the cationic nanoparticle surface, there is no need for nanoparticle disintegration. Instead, miRNA should be detached from the nanoparticles' surface (Fig. 1) (De Jesus and Zuhorn, 2015). Although the cationic nature of formed complexes facilitates the interaction with the anionic cellular membrane, an excessive cationic charge of NLCs can result in the strong binding of miRNAs to nanoparticles, which may hinder the release of miRNAs in the cytosol. Additionally, highly charged nanoparticles exhibit increased interaction with extracellular macromolecules, such as serum proteins (Ponti et al., 2021).

Upon exposure to biological fluids, nanoparticles acquire a new “biological identity” due to the adsorption of biomolecules, including phospholipids and polysaccharides, which leads to the formation of a protein corona, PC (Battaglini et al., 2022). The PC is classified into two layers: the “soft corona”, composed of proteins that are bound to nanoparticles with lower affinity, and the “hard corona” consisting of proteins with higher affinity for nanoparticles (Chen et al., 2019). The formation of a PC can significantly alter the physicochemical properties of miRNA/cNLC complexes, impacting their stability, interactions with cell surface proteins, cellular uptake, and biodistribution (Sebak et al., 2020). This effect was noticed in the significant changes in particle size and surface charge of miRNA/cNLC complexes diluted in serum-free medium (SFM), compared to those obtained after diluting complexes in a medium that contained 5 % and 10 % of fetal bovine serum (FBS). Furthermore, we evaluated the cellular uptake of miRNA/cNLC complexes in two cell lines, 3T3-L1 mouse-derived fibroblasts and MCF-7, which are derived from human epithelial breast cancer. The results demonstrated efficient internalization in both cell lines in a concentration- and time-dependent manner, with a rapid initial uptake within the first 30 min in SFM, whereas the significant reduction in cellular uptake was noticed in the presence of serum as a result of formed PC (Ruseska et al., 2025). Despite these promising results and successful transfection of both animal and human cell lines, we concluded that further investigation is required to assess the effect of PC on nanoparticle-cell interactions and uptake efficiency.

As a potential strategy to address the problem with the formation of PC when cationic miRNA-complexes interact with serum proteins, we hypothesized that decorating (precoating) miRNA/cNLC complexes with serum proteins, such as human serum albumin (HSA), could prevent PC formation and enhance cellular uptake (Fig. 1). Until now, it has been well established that coating nanoparticles with HSA improves their colloidal stability, and what is more, can lead to increased cellular uptake due to receptor targeting (Bolaños et al., 2019; Hyun et al., 2018; Li et al., 2022; Ogawara et al., 2004; Papini et al., 2020; Schubert and Chanana, 2018; Shahapurmath et al., 2025). Therefore, in this study, we investigated the physicochemical properties of HSA-decorated miRNA/cNLC complexes and evaluated their cellular uptake profile, providing insights into their potential as a delivery system for miRNA-based therapeutics compared to uncoated complexes.

## 2 Materials and methods

### 2.1 Materials

For the production of the NLC formulation, octadecylamine (OA) and polyoxyethylene sorbitan monooleate (Tween® 80) were obtained from Sigma-Aldrich Chemie GmbH (Steinheim am Albuch, Germany), while Pluronic F68/Poloxamer 188 was supplied by BASF (Ludwigshafen, Germany). Miglyol® 812 and Precirol® ATO 5 (glyceryl distearate/palmitostearate) were kindly provided by IOI Oleo GmbH (Hamburg, Germany) and Gattefossè Deutschland GmbH (Eschbach, Germany), respectively. Milli-Q® water (Merck Chemicals GmbH, Darmstadt, Germany) was used in all experiments unless stated otherwise.

miRNA mimic transfection control labelled with Cy3 (Fluo-NTC, MW 15,891.5 g/mol), (sequence: UCACAACCUCCUAGAAAGAGUAGA), miRNA mimic negative control (NTC, MW 14,074.31 g/mol) (sequence: UCACAACCUCCUAGAAAGAGUAGA), and miRNA mimic mmu-miR-27a-3p (miRNA-27a, MW 13,454 g/mol) (sequence: UUCACAGUGGCUAAGUUCCGC) were purchased from Dharmacon (GE Healthcare Austria GmbH & Co OG, Vienna, Austria). Human serum albumin (HSA) was obtained from Sigma-Aldrich Chemie GmbH (Steinheim am Albuch, Germany). Nuclease-free water (VWR International, Darmstadt, Germany) was used to prevent nucleic acid degradation. All complexes were prepared under aseptic conditions in a laminar flow hood (Herasafe KS, Thermo Fisher Scientific, Austria). Surfaces were disinfected with RNase AWAY (Sigma-Aldrich Chemie GmbH, Steinheim am Albuch, Germany). Phosphate buffered saline (PBS) was obtained from Gibco (Life Technologies Corporation, Painsley, UK), while zeta water was prepared by adjusting distilled water with sodium chloride (NaCl) to a conductivity of 50 μS/cm.

For cell culture experiments, 3T3-L1 mouse embryonic fibroblasts (CL-173™) were sourced from ATCC (Manassas, VA, USA). 3T3-L1 cells were cultivated in low-glucose (1 g/L) Dulbecco's Modified Eagle Medium (lgDMEM; Gibco, Life Technologies Corporation, Paisley, UK), supplemented with 10 % fetal bovine serum (FBS; Sigma-Aldrich Chemie GmbH, Steinheim am Albuch, Germany), 1 % HEPES buffer (1 M), 1 % l-glutamine (200 nM), and 1 % penicillin/streptomycin (10,000 IU/mL; Gibco, Life Technologies Corporation, Paisley, UK).

The CellTiter96® Aqueous One Solution Cell Proliferation Assay and CytoToxONE™ Homogeneous Membrane Integrity Assay were purchased from Promega (Madison, WI, USA), while Triton X-100 were sourced from Sigma-Aldrich Chemie GmbH (Steinheim am Albuch, Germany).

For metabolic inhibition studies, sodium azide (10 mM), 2-deoxy-d-glucose (30 mM), chlorpromazine (20 μM), EIPA (5-[N-ethyl-N-isopropyl] amiloride) (40 μM), nystatin (120 IU), genistein (300 μM), and dynasore (200 μM) were procured from Sigma-Aldrich Chemie GmbH (Steinheim am Albuch, Germany).

For confocal laser scanning microscopy, Alexa Fluor 488 Phalloidin and DAPI were obtained from Thermo Fisher Scientific (Vienna, Austria), and formalin solution (neutral buffered, 10 %) was ordered from Sigma-Aldrich Chemie GmbH (Steinheim am Albuch, Germany). All chemicals were used as received without further purification.

Furthermore, for the in vitro efficacy study, high-glucose (4.5 g/L) DMEM with phenol red (Gibco, Life Technologies Corporation, Paisley, UK), was used, while insulin, dexamethasone, isobutylmethylxanthine (IBMX), and Oil-Red-O (ORO) were obtained from Sigma-Aldrich Chemie GmbH (Steinheim am Albuch, Germany). 2-propanol, formaldehyde (37 %), and Glycerol (85 %) were sourced from VWR International, LLC (Rosny-sous-Bois, France), Carl Roth GmbH + Co. KG (Karlsruhe, Germany), and Herba Chemosan Apotheker-AG (Vienna, Austria), respectively.

### 2.2 Preparation of cNLC formulation

The cationic NLC (cNLC) formulation was prepared using high-pressure homogenization, one of the most widely employed methods for NLC production (Panda 2K, NS1001L Spezial, GEA Niro Soavi, Parma, Italy), as previously described (Tucak-Smajić et al., 2023). The lipid phase of cNLC comprised 4.365 % (*w/w*) Precirol® ATO 5, 0.485 % (*w/w*) Miglyol® 812, and 0.15 % (*w/w*) OA, while the aqueous phase contained 1.33 % (*w/w*) Tween® 80, 0.67 % (*w/w*) Pluronic® F68, and 93 % (*w/w*) Milli-Q® water.

### 2.3 Preparation of miRNA/cNLC complexes (NLCplexes)

Complexes between miRNAs and cNLCs were prepared using miRNA:OA mass ratios of 1:2.5 and 1:5 (*w/w*). Stock solutions of miRNAs (FluoNTC, NTC, and 27a) were initially prepared in RNase-free water and stored at −80 °C. A working solution of miRNA (1.3 μM) was prepared by diluting the stock solution with RNase-free water. This miRNA working solution was then mixed with a working solution of cNLC (diluted 1:10, *v/v* in RNase-free water). The standard complex (NLCplex) was subsequently diluted with serum-free, phenol-free low-glucose DMEM to achieve final miRNA concentrations of 100 and 200 nM. The mixtures were incubated at room temperature (∼ 23 °C) for 5 min. All stated concentrations refer to miRNA in in vitro experiments.

### 2.4 Decorating the surface of NLCplexes with HSA

The surface of NLCplexes was decorated using HSA. For that purpose, increasing concentrations of a working solution HSA (100 mg/mL) were subsequently added to freshly prepared NLCplexes (1:2.5 and 1:5, *w/w*) and incubated at room temperature. The standard HSA-coated miRNA/cNLC complexes (termed albuplexes) were then diluted with RNase-free water to achieve a final miRNA concentration of 100 nM for particle size and zeta potential measurements, or with low-glucose DMEM for cell culture studies (miRNA concentration in the range of 100–200 nM). This procedure yielded albuplexes with miRNA:cNLC(OA):HSA mass ratios ranging from 1:2.5:10 to 1:2.5:1500 (*w/w/w*) and 1:5:10 to 1:5:1700 (*w/w/w*).

### 2.5 Influence of complexation time on the physicochemical properties of albuplexes

The influence of different incubation times (5 min, 15 min, 30 min, and 60 min) on the preparation of albuplexes was investigated to determine the optimal complexation time. For this purpose, albuplexes (100 nM), with miRNA:cNLC mass ratios of 1:2.5 and 1:5, were prepared as described in Section 2.4. The samples were incubated in Eppendorf tubes for specified periods at a controlled temperature of 25 ± 1 °C using a Thermomixer Comfort (Eppendorf Austria GmbH, Vienna, Austria) at 150 rpm. The physicochemical properties of albuplexes (particle size, polydispersity index (PdI), and zeta potential) were analyzed using a Zetasizer Nano ZS (Malvern Instruments, UK) to evaluate the effect of complexation time.

### 2.6 Stability studies in different dilution media

The influence of dilution media on the physicochemical properties and stability of albuplexes was evaluated using a Zetasizer Nano ZS (Malvern Instruments, UK). Standard albuplexes were prepared in RNase-free water at mass ratios of 1:2.5:1300 and 1:5:1500 (*w/w/w*). The prepared complexes were then diluted in different media: RNase-free water, zeta water, PBS, and serum-free low-glucose DMEM. Following a 5-min incubation after dilution, the final miRNA concentration in the complexes was adjusted to 100 nM, while the HSA concentrations were 31.06 μM and 40.62 μM, respectively.

### 2.7 Characterization studies

#### 2.7.1 Particle size and zeta potential analysis

The hydrodynamic diameter (z-average) and PdI of cNLCs, NLCplexes*,* and albuplexes were measured using a Zetasizer Nano ZS (Malvern Instruments, UK). Particle size analysis of cNLCs was performed in disposable polystyrene cuvettes (PS, semi-micro; Brand GmbH+Co. KG, Germany). For analysis of NLCplexes and albuplexes, UV micro cuvettes (UVette® 220–1600 nm, Eppendorf, Germany) were used. Zeta potential was measured at 25 °C in a disposable folded capillary cell (DTS1070, Malvern Instruments, Herrenberg, Germany).

#### 2.7.2 Atomic force microscopy (AFM)

Surface properties of cNLCs, NLCplexes (1:2.5, *w/w*), and albuplexes (1:2.5:1300, *w/w*) were analyzed using a FlexAFM 5 atomic force microscope (Nanosurf AG, Switzerland), equipped with a C3000 controller. AFM measurements were performed in the air under dry conditions using phase-contrast mode (tapping mode) with a Tap300 Al-G cantilever (BudgetSensors, Bulgaria). For the AFM analysis, cNLCs were diluted 1:100 (*v/v)* in Milli-Q® water. The NLCplexes (1:2.5, *w/w*), and albuplexes (1:2.5:1300, *w/w/w*), were incubated for 5 min and then diluted in RNase-free water to achieve a final miRNA concentration of 100 nM. A 10 μL aliquot of each sample was deposited onto freshly cleaved mica and dried under laminar flow conditions overnight. Data analysis was performed using Gwyddion software (v2.55).

#### 2.7.3 Gel electrophoresis

The presence of HSA on the surface of NLCplexes was further confirmed using gel electrophoresis (SDS-PAGE). Briefly, albuplexes were prepared as previously described, mixed for 1 h at 37 °C, and centrifuged at 4 °C (14 000 rpm). This was followed by removing the supernatant and washing out the unbound HSA using PBS. The samples were then dissolved in Laemmli buffer and denatured for 5 min at 95 °C. Next, the samples were applied to pre-casted polyacrylamide gels (Mini-PROTEAN TGX Gels, Bio-Rad Laboratories, USA) and the gels were run for 45 min. Finally, the gels were stained using the ProteoSilver®Plus Silver Stain Kit (Sigma-Aldrich Co., Darmstadt, Germany) and imaged using the ChemiDoc Go Imaging System (Bio-Rad Laboratories, USA).

#### 2.7.4 UV-VIS spectrophotometry

UV–Vis spectra were recorded using an Eppendorf BioSpectrometer® Kinetic (Eppendorf AG, Germany). The selected wavelength range was 220–800 nm. Measurements were performed in UV–Vis micro cuvettes with a path length of 10 mm at room temperature. The baseline was corrected using MQ water as a blank.

Firstly, the absorbance spectrum of 1 mg/mL HSA dissolved in MQ water was obtained and served as a reference for comparison. Next, NLCplexes (1:2.5 and 1:5 *w/w*), albuplexes (1:2.5:1300, *w/w/w* and 1:5:1500, *w/w/w*), as well as the single components were prepared and diluted using MQ water to a final HSA concentration of 1 mg/mL.

#### 2.7.5 Circular dichroism

CD spectra were recorded on a JASCO J-1500 spectrometer (JASCO, Tokyo, Japan) spectrometer in a quartz cell with an optical path of 1 mm for aqueous solutions of the peptide. The measurements were performed in 200 mL MQ water after diluting all samples to a final HSA concentration of 0.2 mg/mL. Measurements were done at 21 °C, 37 °C, and 65 °C. The signal was converted to delta epsilon [De] (mdeg*M^−1^*cm^−1^) using the following equation:

(1) $$\mathrm{De}=\frac{Q}{C*\mathrm{Nr}*1*32.98}$$

Where Q is the measured ellipticity (in degrees), C is the molar concentration (M), Nr is the number of residues in the peptide, and *l* is the cell path length (cm).

#### 2.7.6 Protein corona

The protein corona analysis was conducted by diluting 1:2.5 and 1:5 albuplexes in low-glucose DMEM supplemented with 10 % FBS. The complexes were mixed with medium and diluted to obtain a final miRNA concentration of 200 nM. The albuplexes were mixed for 1 h at 37 °C and centrifuged at 4 °C (14 000 rpm). This was followed by removing the supernatant and washing out the unbound serum proteins using PBS. Next, the pellet was dissolved in MQ water and the size and charge of the coated complexes were determined using dynamic and electrophoretic light scattering. The measurements were performed using a Zetasizer Nano ZS (Malvern Instruments, UK).

The protein corona was also analyzed by gel electrophoresis. The samples were prepared as previously described and dissolved in Laemmli buffer after centrifugation. The samples were denatured for 5 min at 95 °C and were applied to pre-casted polyacrylamide gels (Mini-PROTEAN TGX Gels, Bio-Rad Laboratories, USA). The gels were run for 45 min and then stained using the ProteoSilver Plus Silver Stain Kit (Sigma-Aldrich Co., Darmstadt, Germany) and imaged using the ChemiDoc Go Imaging System (Bio-Rad Laboratories, USA).

#### 2.7.7 Cytotoxicity studies in 3T3-L1

Cell viability and cytotoxicity of the cNLC formulation, free miRNA, NLCplexes, and albuplexes were assessed using the MTS assay (CellTiter96® Aqueous One Solution Cell Proliferation Assay) and the LDH assay (CytoToxONE™ Homogeneous Membrane Integrity Assay). Following a 4-h incubation of cells with the sample at 37 °C, the supernatant was transferred to a white 96-well plate (Greiner Bio-One GmbH, Germany) for the LDH assay.

After removing the residual sample volume, cells were washed with PBS and prepared for the MTS assay. To each well, fresh serum-free medium and 20 μL of MTS solution were added. After an additional 4-h incubation, absorbance was measured at 490 nm using a UV-VIS plate reader CLARIOstar® Plus (BMG Labtech GmbH, Germany). Cells incubated with culture medium alone served as a control representing 100 % viability, while 0 % viability was determined from cells treated with Triton X-100 (2 %, *w/v*). Cell viability (%) was calculated as the percentage of viable cells relative to untreated controls, using the following Eq. (2):

(2) $$\text{Cell viability}\;\left(\%\right)=\frac{A_{\text{treated cells}}}{A_{\text{untreated cells}}}\times 100$$

For the LDH assay, CytoTox reagent was added to the separated supernatants. After incubation at 37 °C for 20–30 min, stop solution was added to each well. The fluorescence signal, representing LDH release from the culture supernatants, was measured with a UV–Vis plate reader at an excitation wavelength of 560 nm and an emission wavelength of 590 nm. Cell cytotoxicity was expressed as a percentage relative to the maximum LDH release, which was determined using Triton X-100 (2 %, *w/v*). Untreated cells served as the negative control. Cytotoxicity (%) was calculated using the following Eq. (3):

(3) $$\text{Cytotoxicity}\;\left(\%\right)=\frac{F_{\text{test sample}}-F_{\text{negative control}}}{F_{\text{positive control}}-F_{\text{negative control}}}\times 100$$

#### 2.7.8 Uptake kinetics and concentration-dependency

3T3-L1 cells were seeded in 96-well plates (Greiner Bio-One GmbH, Germany) with a seeding density of 8 × 10^3^ cells/well and were cultivated overnight. The transfection was performed by diluting the standard 1:2.5 and 1:5 albuplexes to final miRNA concentrations of 100 and 200 nM. The uptake was traced for 24 h and was evaluated at the following time points: 15 min, 30 min, 1 h, 2 h, 4 h, and 8 h. Before measuring the fluorescence intensity, the complexes were removed, and the cells were washed using pre-warmed PBS. They were resupplied with warm phenol-red-free medium and were permeabilized using 0.1 % Triton X-100. The uptake was quantified by measuring the fluorescence intensity of internalized complexes using a CLARIOstar Plus plate reader (BMG Labtech GmbH, Germany). Untreated cells served as control. The fluorescence intensity obtained from albuplexes applied to wells in which no cells were seeded served as a standard. Furthermore, the obtained data on the uptake of albuplexes was compared to the one previously obtained on the uptake of NLCplexes.

Data obtained from the time series was further used to evaluate the uptake kinetics. For this purpose, the obtained data points for the first 30 min were fitted to a model using GraphPad Prism 10.0. The best fit model was the linear regression model, described by the following equation:

(4) $$\left[y=\mathrm{ax}+b\right]$$

where y is the amount of internalized complexes, a is the slope (the rate constant), x is time, and b is the y-intercept.

#### 2.7.9 Metabolic inhibition studies

To differentiate between endocytic pathways involved in the uptake of albuplexes, different chemical inhibitors were used: chlorpromazine, EIPA (5-[N-ethyl-N-isopropyl] amiloride), genistein, dynasore, and nystatin. 3T3-L1 cells were preincubated with the inhibitors for 30 min. Transfection was performed using albuplexes at both ratios (1:2.5 and 1:5) and the uptake was evaluated after 30 min using the CLARIOstar® Plus plate reader (BMG Labtech GmbH, Germany).

An MTS assay was previously conducted to determine which concentrations of the metabolic and endocytosis inhibitors would be most appropriate for our uptake experiments.

#### 2.7.10 Competitive uptake studies using HSA

To assess the role of surface-bound HSA in the cellular uptake of albuplexes, we conducted a simple experiment of competitive binding. To achieve this, 3T3-L1 cells were seeded in black, glass-bottom, 96-well plates (Greiner Bio-One, GmbH, Germany) and incubated in a cell culture medium supplemented with 10 % HSA for 30 min before transfection. The final concentration of albuplexes was 200 nM. Uptake was evaluated by measuring the fluorescence intensity of FluoNTC using a CLARIOstar® Plus plate reader (BMG Labtech GmbH, Germany).

#### 2.7.11 Confocal laser scanning microscopy

For CLSM studies, 3T3-L1 were seeded in glass-bottom dishes (WillCo Wells B.V., Amsterdam, Netherlands) with a seeding density of 7 × 10^4^ cells and were cultivated overnight. Transfection was performed after diluting 1:2.5 and 1:5 albuplexes using serum-free, phenol-red-free medium to a final concentration of 200 nM. The cells were incubated with the complexes for 30 min and 4 h. In parallel, transfection was also performed with 1:2.5 and 1:5 albuplexes diluted in a medium supplemented with 10 % FBS, to assess the influence of protein corona on the uptake. Furthermore, uptake was evaluated after incubating the cells using cell culture medium supplemented with 10 % HSA.

After each time point, the cells were washed using warm PBS to remove any unbound nanoparticles and subsequently fixed by using 3.7 % formaldehyde. The actin cytoskeleton was stained using Alexa Fluor™ 488 Phalloidin (Thermo Fisher Scientific, Vienna, Austria), and the nuclei were counterstained using DAPI (Thermo Fisher Scientific, Vienna, Austria). The imaging of the samples was done using a confocal laser scanning microscope Leica Stellaris 5 (Leica Microsystems, Germany). The obtained images were analyzed using Fiji 2.9.0.

#### 2.7.12 In vitro efficacy studies in 3T3-L1 preadipocytes

3T3-L1 preadipocytes were cultivated in a proliferation medium consisting of low-glucose DMEM, supplemented with 10 % FBS, 1 % l-glutamine, 1 % HEPES, and 1 % penicillin/streptomycin. Cells were seeded in 96-well plates (Greiner Bio-One GmbH, Frickenhausen, Germany) at a density of 7 × 10^3^ cells/well and incubated 24 h before transfection.

For transfection, cells were divided into two groups:

1. The control group consisted of single substances used for NLCplex and albuplex preparation: cargo-free cNLC, free miRNA-27a (200 nM), free miRNA-NTC (200 nM), and free HSA (81.2 μM).
2. The experimental group composed of NLCplexes (miRNA-27a:cNLCs and miRNA-NTC:cNLC) at mass ratios 1:2.5 and 1:5 (miRNA concentration 200 nM), as well as albuplexes (miRNA-27a:cNLC:HSA and miRNA-NTC:cNLC:HSA) at mass ratios 1:2.5:1300 and 1:5:1500.

After 4 h of incubation, a transfection medium composed of low-glucose DMEM supplemented with 20 % FBS, 1 % l-glutamine, and 1 % HEPES was added to wells, adjusting the final miRNA concentration was 100 nM, as explained in (Tucak-Smajić et al., 2023).

The following day, the medium was removed, and cells were washed with PBS. To induce differentiation of preadipocytes to mature adipocytes, an induction medium containing high-glucose DMEM, 10 % FBS, 1 % l-glutamine, 1 % HEPES, 1 % penicillin/streptomycin, 1 % IBMX (500 μM), 0.1 % insulin (10 μg/mL), and 0.1 % dexamethasone (1 μM) was added. On days 2 and 4 post-transfection, the medium was replaced with a differentiation medium composed of high-glucose DMEM, 10 % FBS, 1 % l-glutamine, 1 % HEPES, 1 % penicillin/streptomycin, and 0.05 % insulin.

On day 6 of differentiation, mature adipocytes were stained with Oil Red O (ORO) to visualize intracellular lipid droplets. Cells were fixed in 10 % formaldehyde (1:10 in PBS) for 1 h, followed by washing with MQ water and incubation with 60 % (*v*/v) 2-propanol (in MQ water) for 5 min. After complete air-drying of wells, cells were stained with ORO working solution for 10 min and repeatedly washed with MQ water. To prevent sample drying, cells were covered with 50 % (v/v) glycerol (in MQ water).

For control conditions, differentiated cells were treated as described above, while an additional control group consisted of undifferentiated cells maintained in a proliferation medium only.

The degree of differentiation was assessed by staining lipid droplets and imaging under a Leica DMIL microscope Type 090–135.002 (Leica Microsystems GmbH, Vienna, Austria) equipped with a Canon EOS 70D digital camera (Canon GmbH, Vienna, Austria).

The semiquantitative evaluation of accumulated ORO dye was performed using a CLARIOstar® Plus plate reader (BMG LABTECH, Ortenberg, Germany) by measuring absorbance at 500 nm in matrix scan mode (25 × 25). Glycerol 50 % (v/v) was used as a blank in empty wells.

### 2.8 Statistical analysis

In the presented paper, numerical values are presented as *mean ± SD (n = x).* Statistical analysis was done using a *t*-test and ANOVA. *P* values of 0.05 or less were considered statistically significant (*p* < 0.05 (*), *p* < 0.01 (**), *p* < 0.001 (***), and *p* < 0.0001 (****)).

## 3 Results and discussion

### 3.1 Particle size and surface charge of cNLCs, NLCplexes, and albuplexes

High-pressure homogenization (HPH) was chosen for the preparation of cNLCs due to its simplicity, reproducibility, scalability, solvent-free operation, and ability to produce stable nanoparticles with narrow size distribution (Tapeinos et al., 2017), making it particularly suitable for downstream biological applications. In our previous study, we developed cNLCs as delivery systems for miRNA based on electrostatic adsorption onto the particle surface, aiming to improve cellular uptake of miRNA-27a (Tucak-Smajić et al., 2023) since less than 2 % of nucleic acids encapsulated in LNs escape from the endosome to reach the cytosol (Spadea et al., 2022). The optimized cNLC formulation, formulated using physiologically relevant lipids and surfactants that are generally recognized as safe (GRAS), demonstrated a particle size of around 115 nm and a highly cationic zeta potential (Table 1), with excellent physicochemical stability over a 9-month period (Tucak-Smajić et al., 2023). After two years of storage at 5 ± 3 °C, the particle size slightly increased to 156.67 ± 1.46, with a PdI of 0.243 ± 0.009, while the ZP decreased to 37.60 ± 1.32 mV, indicating excellent long-term stability.

**Table 1:** Particle size and zeta potential of cargo-free cNLC formulation, and NLCplexes at mass ratios of 1:2.5 and 1:5 (*w/w*).

	***Cargo-free cNLC***	***NLCplexes***	
		1:2.5 (*w/w*)	1:5 (*w/w*)
*z-ave (nm)*	116.4 ± 2.9	139.7 ± 1.7	120.7 ± 0.5
*PdI*	0.194 ± 0.011	0.222 ± 0.011	0.185 ± 0.020
*Zeta potential (mV)*	43.80 ± 1.40	34.50 ± 0.65	46.80 ± 0.66

MiRNA/cNLC complexes (NLCplexes) were subsequently prepared at miRNA:cNLC mass ratios of 1:2.5 and 1:5 (*w*/w), resulting in particle sizes ranging from 120 to 140 nm and zeta potential values exceeding 28 mV (Table 1), which are considered favourable for efficient cellular uptake (Tucak-Smajić et al., 2023). At the 1:5 ratio (*w*/w), miRNA molecules primarily adsorb onto the surface of cNLCs, but the overall number of cationic lipids exceeds the amount of nucleic acid. Consequently, not all positive charges are neutralized, resulting in a net positive surface charge. This is consistent with established observations in lipoplex formation, where a slight-to-moderate excess of cationic lipids is necessary to ensure complete encapsulation or “burial” of nucleic acids within the complex (Elouahabi and Ruysschaert, 2005).

Cellular uptake studies in 3T3-L1 cells confirmed rapid, concentration- and time-dependent internalization of NLCplexes, with pronounced uptake within the first 30 min. However, the presence of serum proteins markedly influenced uptake efficiency, likely due to PC formation altering the surface characteristics of the complexes (Ruseska et al., 2025). This highlights the complexity of NLCplex behaviour in biological environments, as not only proteins but also various biomolecules (e.g., vitamins, ions, and growth factors) present in cell culture media can modify their surface properties (Bhattacharjee, 2016).

**Fig. 2:**
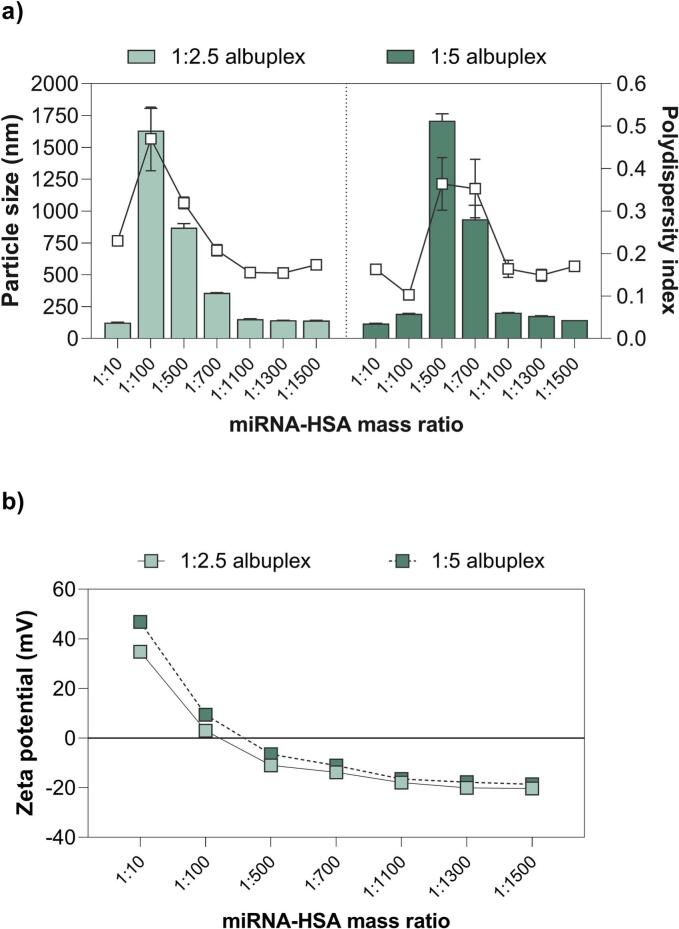
Effect of HSA addition on (a,) particle size and size distribution, and (b) surface charge of *NLCplexes* (100 nM) at a mass ratio of 1:2.5 and 1:5. The mass ratio of miRNA (from the complex) to HSA ranged from 1:10 to 1:1500 (*w/w*).

These modifications can further influence nanoparticle stability, interactions with cell surface receptors, cellular uptake efficiency, and ultimately, drug release dynamics. Therefore, to improve the stability and cellular internalization of the NLCplexes under physiological conditions, we decorated their surface by the gradual addition of HSA (Fig. 1). The impact of HSA addition on NLCplex particle size, size distribution, and zeta potential at mass ratios 1:2.5 and 1:5 was assessed by DLS and ELS, as shown in Fig. 2. The impact of incubation time on the complexation is presented in Supporting Information: Table S1.

In the beginning, the gradual addition of HSA to the pre-formed NLCplexes at mass ratios 1:2.5 and 1:5 led to an increase in particle size, which peaked at 1:2.5:100 and 1:5:500 miR:OA:HSA mass ratios, respectively (Fig. 2a). The addition of HSA to NLCplexes, starting from the miRNA:HSA ratio of 1:100 (w/w) and 1:300 (w/w), was sufficient to only partially cover NLCplex surfaces. This partial coverage caused HSA molecules to simultaneously adsorb onto multiple NLCplexes, acting as “molecular bridges” that linked particles into large aggregates with sizes exceeding 1 μm. In this case, the DLS technique detected micron-sized assemblies. A similar phenomenon was reported for serum proteins interacting with colloidal nanoparticles, where incomplete corona formation promoted interparticle cross-linking and aggregation (Tirado-Miranda et al., 2003).

Following this, at a miRNA:HSA mass ratios above 1:700 (w/w) (Fig. 2a), the particle size stabilized between 140 and 145 nm, and the PdI values remained below 0.2, indicating successful coating of the NLCplexes with HSA. As the HSA concentration increased beyond this threshold (≥ 1:700 w/w), it appears that the NLCplex surfaces reached full saturation, forming a uniform, multi-layered “hard” corona. The resulting steric hindrance between the formed *albuplexes* prevented further particle–particle interactions, which restored hydrodynamic diameter to ∼140 nm. A similar steric stabilization effect at higher BSA-to-cationic-ligand functionalized gold nanorod ratios was observed in a study of (Dominguez-Medina et al., 2016), where a uniform BSA coating stabilized the particles and prevented aggregation, which was otherwise observed at lower protein: nanoparticle ratio due to BSA unfolding.

Concurrently, the ZP gradually decreased due to the anionic nature of HSA, from 34.5 mV to −20.3 mV for 1:2.5 NLCplexes, and from 46.8 mV to −18.6 mV for the 1:5 NLCplexes (Fig. 2b). Since no significant changes in particle size and surface charge were observed above the mass ratios of 1:1300 for 1:2.5 NLCplexes and 1:1500 for 1:5 NLCplexes, these albuplexes were selected for further studies.

Although the particle size between NLCplexes and albuplexes was similar, the ZP shifted from highly cationic to anionic values. The cationic nature of complexes possesses advantages such as interaction with anionic cell membrane, and rapid uptake in serum-free medium. However, we previously observed a concentration-dependent decrease in cell viability in the 3T3-L1 cell line (Ruseska et al., 2025; Tucak-Smajić et al., 2023). In contrast, the negative charge of albuplexes is expected to reduce potential toxicity compared to highly cationic nanoparticles, enhance particle stability by promoting electrostatic repulsion, and improve cellular uptake by receptor-mediated endocytosis, enabling targeted delivery. Additionally, the formation of a stable HSA corona may prevent the nonspecific adsorption of serum proteins and the formation of an undesirable protein corona, potentially improving systemic circulation time and reducing premature immune system recognition.

The stability of albuplexes in different dilution media is shown in Supporting Information: Fig. S2. While the physicochemical properties of albuplexes were preserved in RNase-free water, PBS and low-glucose DMEM induced noticeable changes, especially at higher mass ratios, likely due to ionic interactions and medium composition. In addition, the AFM analysis (Supporting Information: Fig. S3) confirmed that miRNA complexation and HSA coating modified nanoparticle surface morphology. Albuplexes exhibited smoother, more uniform surfaces, indicating improved stability and potential suitability for biological applications. Although AFM provides indirect information on coating through particle size and surface morphology, direct visualization will be addressed in future studies using cryo-TEM. Based on these results, optimized conditions included a 5-min incubation and preparation in RNase-free water for subsequent studies to ensure efficient formulation with minimal preparation time.

### 3.2 UV/VIS spectrophotometry

**Fig. 3:**
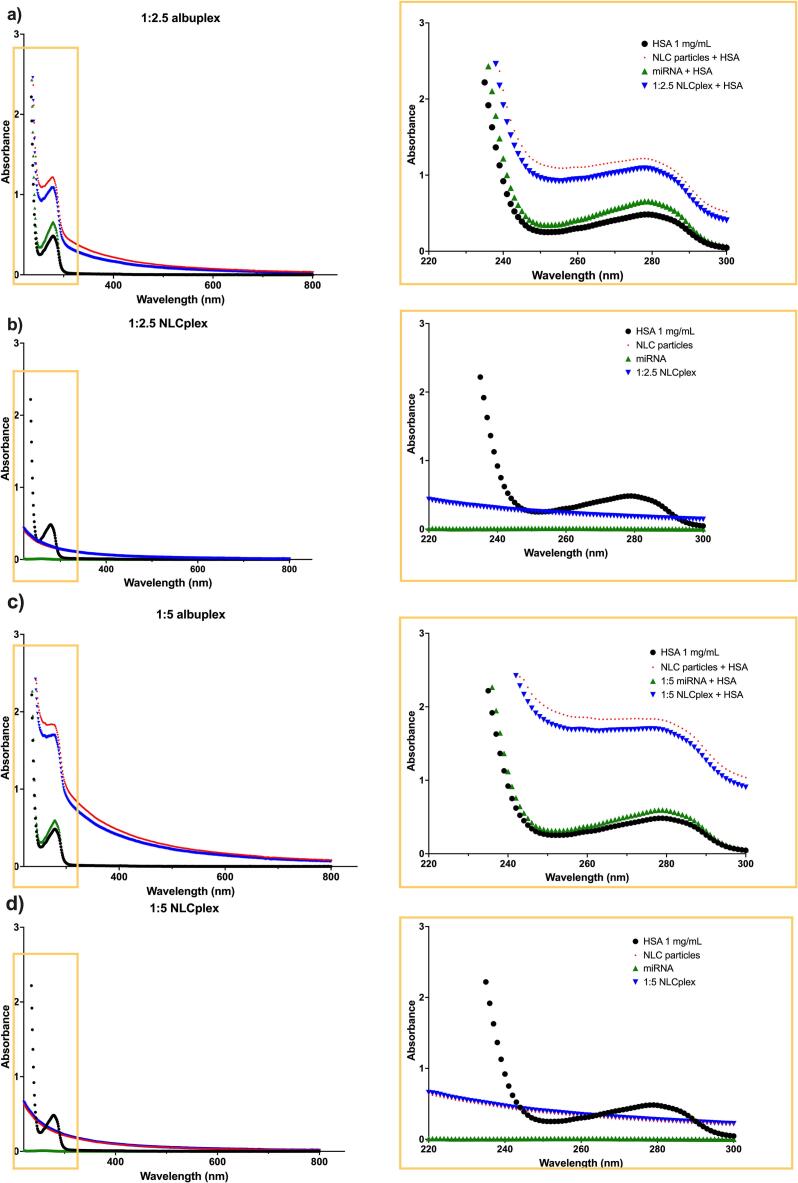
UV/Vis spectra recorded for a) 1:2.5 albuplexes, b) 1:2.5 NLCplexes, c) 1:5 albuplexes, and d) 1:5 NLCplexes. The spectra of all formulations were compared to those of free HSA, free miRNA, and uncomplexed NLCs. The region of interest, where we expect HSA absorbance maxima, is presented in the zoomed-in images.

The UV/Vis absorption measurement is a simple and effective method for studying protein-nanoparticle interactions and monitoring associated conformational changes. The UV/Vis spectra of HSA, NLCplexes, and albuplexes are shown in Fig. 3. The spectra were obtained from 220 to 800 nm, with HSA absorption maxima at 220 nm and 278 nm, due to its framework conformation and π → π* transitions of aromatic amino acids (such as Tyr, Phe, Trp), respectively (Baig et al., 2019; Qais et al., 2020).

As seen in the Fig. 3, the peaks were retained in the albuplex formulations, as well as complexes between the NLCs and albumin (without miRNA), confirming the presence of albumin on the surface of the particles. While the absorption peak at 220 nm remained largely unchanged, we observed a more intense peak at 278 nm (hyperchromism). This pattern was observed for both 1:2.5 and 1:5 formulations (Fig. 3a,c). The observed effect might be explained by changes in the microenvironment of the tryptophan residues in HSA, induced by the electrostatic interactions between the protein and lipid core. This might lead to the amino acid residues being extended to the aqueous environment (Baig et al., 2019; Mohammadi et al., 2017). It was worth to mention that we also observed a miRNA absorbance (∼ 0.02). However, due to the substantially higher absorbance of HSA and the albuplexes in this region, it is challenging to clearly represent the miRNA absorbance in the combined spectra (Fig. 3b). Therefore, a separate graph showing the absorbance of the naked miRNA was added in Supplementary material (Fig. S4). Nevertheless, the rise in intensity might also occur due to the protein corona effect, which would lead to a local enrichment of HSA, together with the formation of larger complexes. Indeed, this was confirmed by DLS – coating the NLCplexes with HSA led to increased size compared to the uncoated formulations. Furthermore, we evaluated whether or not any conformational changes in the structure of HSA occur when it is complexed with miRNA. The obtained data indicate that there is a modification of the protein's conformation, given that the peak's intensity was slightly increased at 280 nm (Fig. 3a,c). This could also be the result of structural alterations due to electrostatic interactions between HSA and miRNA (Botti et al., 2022a, Botti et al., 2022b).To analyze this matter further, we conducted CD spectroscopy.

### 3.3 Circular dichroism

The far-UV CD spectra of proteins depend on their secondary structure and reveals information about the structural class of the protein. Generally, all α-helical proteins are characterized by an intense negative band with two peaks (at 208 and 222 nm), and a positive band at around 190 nm. b-proteins, on the other hand, demonstrate significantly weaker spectra, with a negative band at 210–225 nm, and a positive band at 190–200 nm. Lastly, unordered proteins are characterized by a strong negative band at 195–200 nm, and a weaker band between 215 and 230 nm (Martin and Schilstra, 2008).

**Fig. 4:**
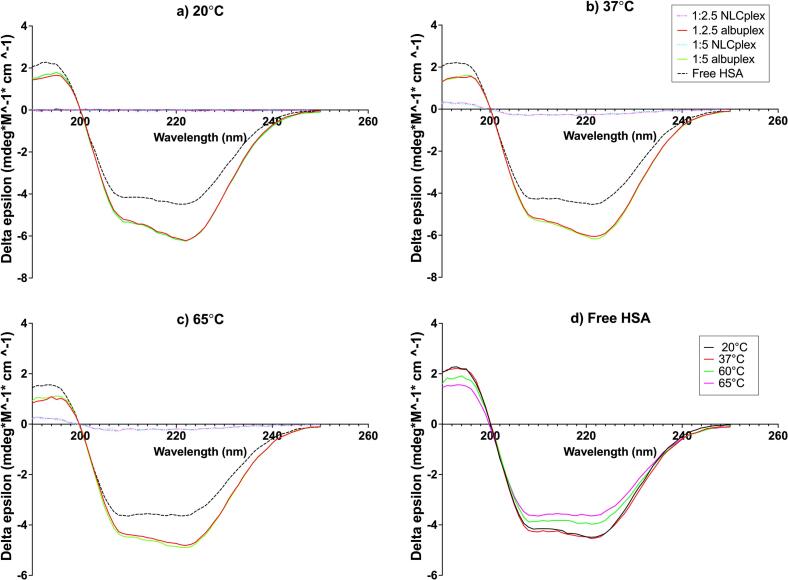
Far-UV CD spectra obtained from albuplexes, NLCplexes, and free HSA, at a) 20 °C, b) 37 °C, and c) 65 °C. The CD spectra obtained from free HSA are also presented separately in d.

The obtained far-UV CD spectra of albumin at room temperature (20 °C) exhibited a strong negative band with two peaks – one at 208 nm, and a second one at 220 nm, indicating a predominant α-helical structure, typical for albumin (Gao et al., 2004). Upon complexation with 1:2.5 and 1:5 NLCplexes and obtaining albuplexes, the spectral shape remained largely unchanged. However, the spectral intensity increased slightly after complexation, with changes at 208/222 nm (Fig. 4a). By using BeStSel we estimated the secondary structure content (presented in Table 2), and these results indicate that after binding to NLCplexes at room temperature, the α-helicity of albumin marginally increases. Furthermore, we observed an increase in anti-parallel and parallel b-sheets, together with less unordered regions of the protein.

**Table 2:** Estimated secondary structure of free HSA and complexed with 1:2.5 and 1:5 NLCs (in albuplexes), at various temperatures.

*Secondary structure (%)*	*Human serum albumin (HSA)*			*1:2.5 albuplex*			*1:5 albuplex*		
	*20* *°C*	*37* *°C*	*65* *°C*	*20* *°C*	*37* *°C*	*65* *°C*	*20* *°C*	*37* *°C*	*65* *°C*
*Helix*	27.5	25.3	21.1	28.4	27.4	22.4	28.3	27	22.2
*Anti-parallel*	11.5	11.5	10.9	15.5	15	14.2	15.4	15.3	14.2
*Parallel*	9.5	11.4	11.4	11.6	12.3	12.9	12.2	12.2	12.5
*Turn*	13	12.6	13.8	11	10.9	12.4	10.9	11	12.1
*Others*	38.6	39.9	42.7	33.5	34.5	38.1	33.3	34.5	39

To further analyze the potential effect that NLCplexes might have on albumin's structure, we conducted thermal stability studies, where we heated up the samples to 37 °C and 65 °C during the measurement, given that HSA can undergo thermal denaturation (Moriyama et al., 2008). The upper temperature limit was set to 65 °C in order to avoid potential melting of the lipid core and destabilization of the nanoparticles. CD analysis at 37 °C revealed that the spectra of albumin and albuplexes remained largely unchanged in shape and intensity compared to 20 °C (Fig. 4b). In the case of native albumin, a slight decrease in α-helicity was observed (only 2 %), whereas in albuplexes the percentage of albumin α-helices was preserved at values similar to those measured for native albumin at 20 °C. At 65 °C we observed a decrease in the spectra intensity, likely owing to thermal unfolding of the protein and loss of α-helicity (Fig. 4c). Nevertheless, the observed percentage of α-helices and b-sheets remained slightly higher for albuplexes compared to native albumin.

The observed effect that NLCplexes had on the secondary structure of HSA after forming albuplexes indicates that the interaction between the two components leaves the structure of the protein largely intact. Indeed, data suggest that fatty acid (FA) binding can stabilize the conformation of HSA and even alter the protein's stability against thermal denaturation (Frahm et al., 2013; Gao et al., 2004). Physiologically, HSA works as a carrier for FAs, with up to eight FA binding sites. Literature suggests that the binding is driven through hydrophobic and van der Waals interactions, which result in a more stable HSA conformation (Linciano et al., 2022). However, published data also demonstrate that after interacting with cationic or negatively-charged lipids, HSA is destabilized and the secondary structure is lost (Charbonneau et al., 2009; Charbonneau and Tajmir-Riahi, 2010; Tretiakova et al., 2022). In the case of cationic lipids (such as 1,2-dioleoyl-3-trimethylammonium-propane - DOTAP and dimethyldioctadecylammonium bromide - DDAB), Charbonneau et al. suggest that the binding between the lipids and the protein occurs by hydrophobic interactions, which might induce structural changes in HSA that lead to unfolding and exposure of hidden hydrophobic regions (Charbonneau et al., 2009). In the case of negatively-charged lipids, unfolding was observed after the interaction of HSA with the most polar lipid (Tretiakova et al., 2022).

To our knowledge, the preserved structural integrity of a protein in the presence of cationic lipids (in the form of nanostructured lipid carriers), has not been reported to date. We believe that the HSA-NLCplex complexation is most likely driven by electrostatic interactions, a process previously reported for gold nanoparticles (Saikia and Das, 2023), where protein stabilization was also observed (Yang et al., 2019). The amine group of octadecylamine, located on the surface of NLCplexes, can form hydrogen bonds with the carboxyl or hydroxyl groups of amino acid chains in HSA. Additionally, the hydrophobic interactions between the alkyl chain of octadecylamine and the hydrophobic pockets within HSA can contribute to the binding. These interactions can lead to electrostatic and hydrophobic stabilization of the protein, locking it into a more ordered (helical) state (Ashraf et al., 2023; Lee et al., 2024; Thomas et al., 2014).

### 3.4 Protein corona – effect on the physicochemical properties of albuplexes

Exposure to a biological environment leads to the nanoparticles acquiring a new biological identity due to alterations in their surface chemistry. These changes arise from the adsorption of biomolecules – such as proteins, polysaccharides, and phospholipids – onto the nanoparticle surface. Given the abundance of proteins in biological fluids, this newly formed surface layer is commonly referred to as the protein corona (PC). The formation of the protein corona can significantly influence the physicochemical properties of nanoparticles, including their stability, interactions with membrane proteins, cellular uptake, and drug release. Therefore, by modulating protein corona formation, it is possible to tailor the in vivo behaviour of nanoparticles and enhance their therapeutic efficacy.

**Fig. 5:**
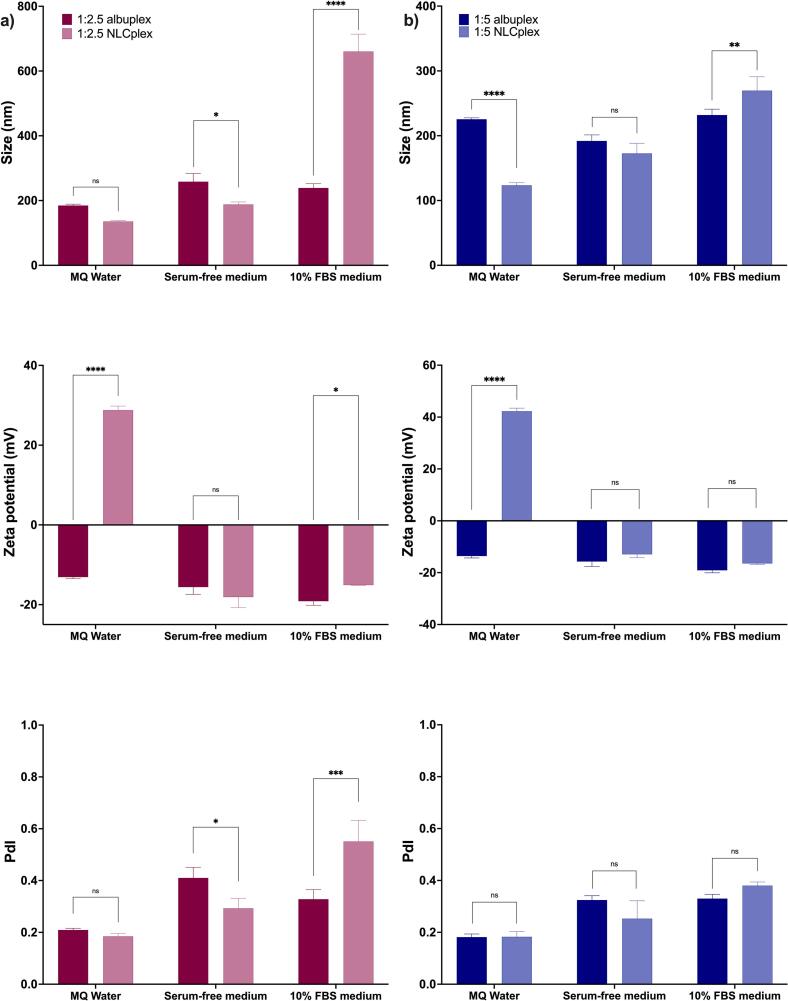
Analysis of protein corona formation on a) 1:2.5 NLCplexes and 1:2.5 albuplexes, and b) 1:5 NLCplexes and 1:5 albuplexes. The protein corona was analyzed in terms of size, charge, and PdI. Data were analyzed using two-way ANOVA, with *p*-values of 0.05 or less considered statistically significant (*p* < 0.05 (*), *p* < 0.01 (**), *p* < 0.001 (***), and *p* < 0.0001 (****)).

The protein corona significantly affected the physicochemical properties and cellular uptake of NLCplexes, as demonstrated in our previous study (Ruseska et al., 2025). To overcome the poor stability and low internalization observed after serum protein exposure, we coated the NLCplexes with HSA. The resulting albuplexes were prepared at 1:2.5 and 1:5 ratios and diluted in either MQ water, serum-free medium (SFM), or medium supplemented with 10 % fetal bovine serum (10 % FBS medium). These albuplexes were then characterized for size, zeta potential (ZP), and polydispersity index (PdI), while the protein corona composition was assessed via gel electrophoresis. The data are presented in Fig. 5.

When diluted in MQ water, the 1:2.5 albuplexes (Fig. 5a) exhibited a negative surface charge (ZP of −13.1 ± 0.3 mV), in contrast to the corresponding NLCplexes, which were positively charged (ZP of 28.8 ± 1 mV). The hydrodynamic diameter of the albuplexes was 184.8 ± 4 nm, and that of NLCplexes was 135.6 ± 3 nm. A similar trend was observed for the 1:5 formulation (Fig. 5b), with albuplexes showing a ZP of −13.6 ± 0.7 versus 42.3 ± 1 mV for NLCplexes, and a significantly larger size (225.4 ± 2 nm vs. 123.5 ± 4 nm, p < 0.001). In both formulations, PdI values were below 0.2, indicating a monodisperse system. The increased size, together with the switch of the surface charge from positive to negative, are indicators of HSA being adsorbed on the surface of the complexes (Rampado et al., 2020).

When SFM was used as the dilution medium, both 1:2.5 albuplexes and NLCplexes exhibited a negative surface charge (−15.5 ± 1 mV and − 18.1 ± 2 mV, respectively) along with a slight increase in particle size. The hydrodynamic diameter of the albuplexes was 258.3 ± 25 nm, whereas that of NLCplexes was 188.3 ± 7 nm. A similar pattern was observed for the 1:5 formulation, where the albuplexes had a ZP of −15.7 ± 1 mV, and the corresponding NLCplexes showed a ZP of −12.9 ± 1 mV. The difference in particle size between albuplexes and NLCplexes in SFM at this ratio was minimal, with measured diameters of 191.9 ± 9 nm and 172.8 ± 21 nm, respectively. Furthermore, 1:5 albuplexes had a lower propensity for agglomeration compared to 1:5 NLCplexes. The polydispersity index increased above 0.3 for both formulations at both ratios, with the difference in PdI between 1:2.5 albuplexes and NLCplexes being significant (p < 0.05). Although serum-free low glucose medium does not contain any proteins, its ionic strength is higher compared to MQ water, and can compress the diffuse layer of the nanoparticles, leading to their agglomeration – a phenomenon which we observed for both formulations at the two ratios (Jonassen et al., 2012; Schachner-Nedherer et al., 2019). Comparing the 1:2.5 and 1:5 formulations, the 1:5 albuplexes appeared more stable in SFM, likely due to their higher albumin content, which resulted in a more negative surface charge and improved colloidal stability (Guerrini et al., 2018).

The presence of serum proteins had a pronounced effect on the size of 1:2.5 NLCplexes compared to 1:2.5 albuplexes (p < 0.001), with measured hydrodynamic diameters of 660.5 ± 53 nm and 239.0 ± 12 nm, respectively. This effect was also reflected in the surface charge: the ZP of NLCplexes was −15.1 ± 0.1 mV, while albuplexes exhibited a more negative charge of −19.1 ± 1 mV. Similarly, the 1:5 formulation demonstrated improved colloidal stability. The measured diameters of 1:5 NLCplexes and albuplexes were 269.6 ± 21 nm and 232 ± 9 nm, respectively. Both were negatively charged with ZP values of −16.4 ± 0.3 mV for NLCplexes and − 19.1 ± 0.9 mV for albuplexes. The PdI increased significantly for 1:2.5 NLCplexes in the presence of serum (p < 0.001), exceeding 0.5 and indicating strong aggregation. In contrast, both 1:5 albuplexes and NLCplexes maintained PdI values around 0.3, suggesting better colloidal stability. By using HSA as a dysopsonin before incubating NLCplexes in serum-supplemented medium, it is possible to avoid the surface attachment of free serum-proteins (Guerrini et al., 2018). The observed enhanced stability of albuplexes – particularly at the 1:5 ratio – can be attributed to the electrostatic repulsion and steric hindrance provided by the higher amount of albumin on the surface, which helps preserve the integrity of the diffuse layer surrounding the particles (Rampado et al., 2020).

Data obtained from SDS-PAGE (Supplementary material: Fig. S5) after coating the NLCplexes with HSA demonstrate the characteristic HSA (Mw 66 kDa) band between 55 and 75 kDa. The 66 kDa HSA band was also the most prominent band in all the other tested conditions (MQ water, SFM, and 10 % FBS medium) (Supplementary material: Fig. S6). Additionally, there was no change in this band after incubation in 10 % FBS medium, indicating that only trace amounts of BSA are adsorbed on the surface of albuplexes.

### 3.5 Cell viability and cytotoxicity

Cell viability and cytotoxicity of the cNLCs, free miRNA-FluoNTC, NLCplexes (1:2.5 and 1:5, *w/w*), and albuplexes (1:2.5:1300 and 1:5:1500, *w/w/w*) were assessed using the MTS and LDH assays.

**Fig. 6:**
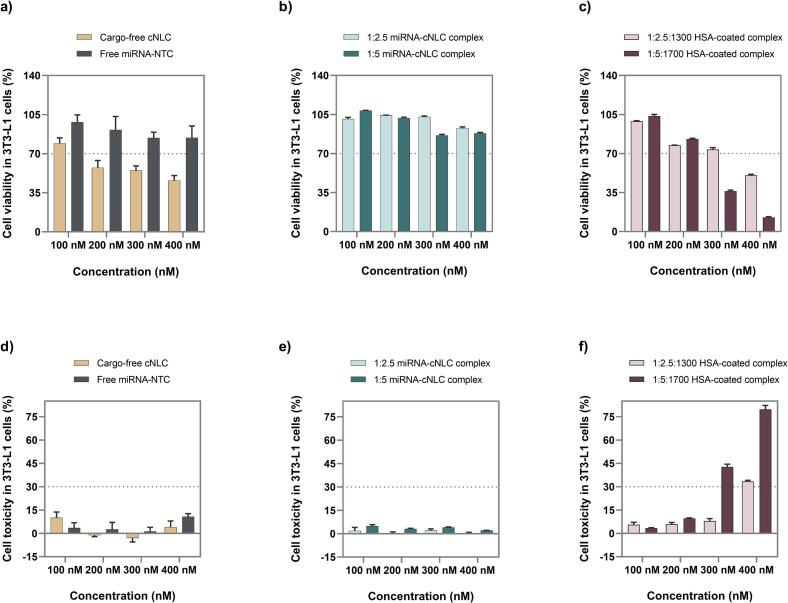
Cell viability (a—c) and cytotoxicity (d—f) of cargo-free cNLC, free miRNA-FluoNTC, NLCplexes at mass ratios of 1:2.5 and 1:5, and albuplexes at mass ratios of 1:2.5:1300 and 1:5:1500 (miRNA concentration: 100–400 nM).

The MTS and LDH assays revealed that free miRNA-FluoNTC, at concentrations ranging from 100 to 400 nM, exhibited no significant cytotoxicity to 3T3-L1 to cells (Fig. 6a,d), with cell viability maintained between 84 and 98 %, and cell toxicity below 15 %. These results are consistent with our previous studies, which assessed the cytotoxic profiles of miRNA-27a and NTC at concentrations ranging from 25 to 100 nM, as well as miRNA-FluoNTC (100–400 nM) (Ruseska et al., 2025; Tucak-Smajić et al., 2023). The high viability can be attributed to the physicochemical properties of miRNAs, including their small size, hydrophilic nature, and negative charge, which leads to electrostatic repulsion between the molecules and the negatively charged cell membrane, thereby limiting their cellular uptake (Hou et al., 2021).

Although a dose-dependent decrease in cell viability was noticed for cargo-free cNLCs, with a reduction from around 79.3 % to 45.9 % (Fig. 6a), the complexation with miRNAs to form NLCplexes at mass ratios 1:2.5 and 1:5 reduced the toxic profile of cNLCs. In all tested concentrations, the cell viability of NLCplexes was above 85 % (Fig. 6b,e). These results can be explained by the presence of phosphate groups in miRNA, which neutralize some of the positive charges on the OA molecules, thereby reducing electrostatic interactions with cellular membranes. These findings align with previous research on the toxicity of nucleic acid/cationic liposome complexes (Fraga et al., 2008, Fraga et al., 2011; Isar et al., 2020).

Furthermore, albuplexes prepared at mass ratios of 1:2.5:1300 and 1:5:1500, with final miRNA concentrations ranging from 100 to 400 nM, also exhibited a dose-dependent cytotoxic profile (Fig. 6c, f). Generally, HSA is considered a very safe compound known to reduce toxicity and prolong the half-life of coated nanoparticles (Ashraf et al., 2023; Svensson et al., 2014). In this case, however, the observed cytotoxicity is likely attributed to the higher content of cationic lipid in the formulation, combined with the increased albumin concentration. Under these conditions, the coated nanoparticles may induce alterations in osmotic pressure and membrane destabilization, as evidenced by the LDH assay. As cell viability above 70 % is generally considered acceptable, we selected albuplexes that contain 100 and 200 nM of miRNA as safe DDS for subsequent cell culture studies.

### 3.6 Uptake kinetics and concentration-dependency

The cellular uptake of nanoparticles is a critical determinant of their therapeutic efficacy and intracellular fate. Nanoparticle internalization typically occurs via endocytic pathways, including clathrin-mediated endocytosis, caveolae-mediated endocytosis, and macropinocytosis. The route of entry significantly influences the intracellular trafficking and eventual bioavailability of the cargo. In our previous study, we investigated the uptake behaviour of NLCplexes in 3T3-L1 and MCF-7 cells in terms of concentration and time-dependency, together with the underlying mechanism that drive the internalization (Ruseska et al., 2025). Our data suggested that the uptake was time- and concentration-dependent, exhibiting a zig-zag pattern over 24 h with rapid uptake observed within the first 30 min. Furthermore, metabolic inhibition and colocalization studies suggested that most likely clathrin-mediated endocytosis and macropinocytosis are involved in the uptake of NLCplexes.

**Fig. 7:**
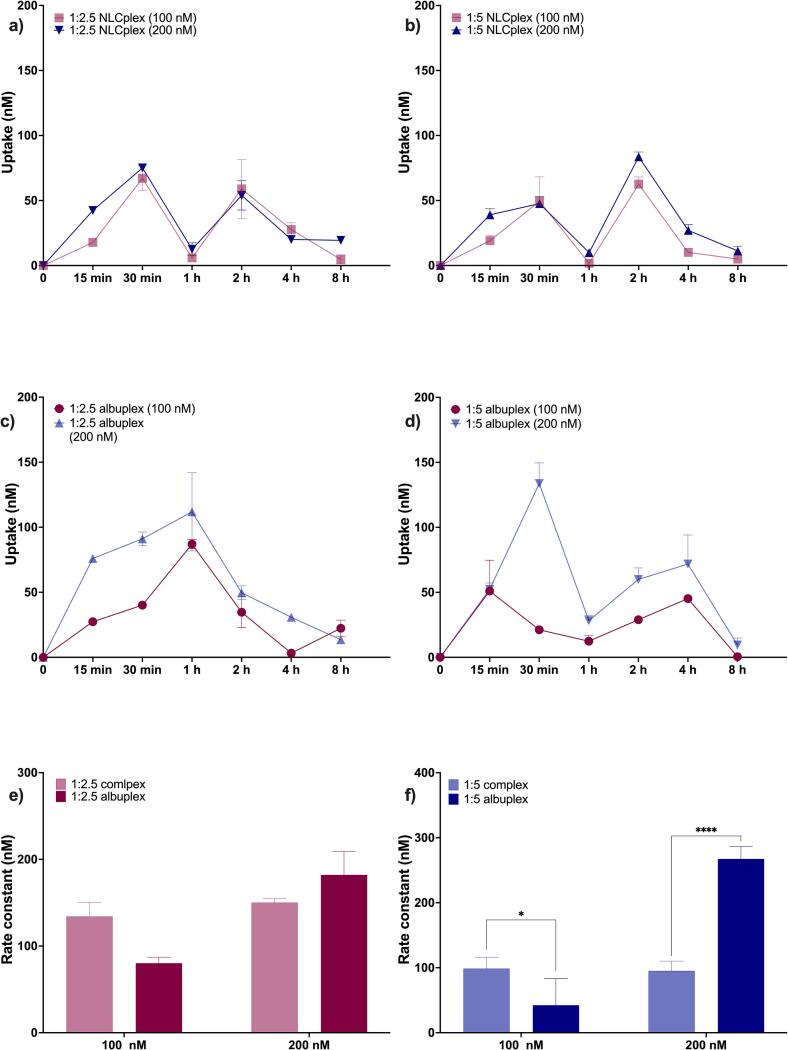
Cellular uptake studies in 3T3-L1 cells conducted for 1:2.5 and 1:5 NLCplexes (a and b) and 1:2.5 and 1:5 albuplexes (c and d). The uptake was characterized in terms of concentration- and time-dependency. Furthermore, the time series were used as a basis for calculating the constants of binding using a linear regression model using GraphPad Prism 10. Data were analyzed using two-way ANOVA, with *p*-values of 0.05 or less considered statistically significant (*p* < 0.05 (*), *p* < 0.01 (**), *p* < 0.001 (***), and *p* < 0.0001 (****).

Based on their biocompatibility, we investigated the uptake of two concentrations (100 and 200 nM) of albuplexes and compared them to the corresponding NLCplexes in the span of 8 h. We found out that the HSA coat influenced the uptake kinetics across both tested ratios and concentrations (Fig. 7).

Specifically, the uptake of 1:2.5 albuplexes was slower compared to the respective NLCplexes (Fig. 7a,c). The uptake of 1:2.5 NLCplexes took 30 min, and in this time the cells had taken up more than 50 % of both 100 and 200 nM formulation. Compared to this, the uptake of 1:2.5 albuplexes took 1 h to reach a plateau, with approximately 90 % (87 ± 3.5 nM) being internalized from the 100 nM formulation, and 55.9 % (111.8 ± 30 nM) being taken up from the 200 nM. The slower uptake of albuplexes might be due to electrostatic repulsion between the negatively charged nanoparticles and the similarly charged extracellular matrix and cell membrane. The electrostatic repulsion may contribute to an energetic barrier that impedes internalization (Engin et al., 2017; Olivieri et al., 2024; Walters et al., 2015; Yadav and Chaudhary, 2021). This effect is reflected in the lower rate constants obtained by fitting the uptake data to a linear regression model using Eq. (4) (Fig. 7e). The calculated rate constants were as follows: 134.2 ± 16.1 nM and 150.2 ± 4.6 nM for 100 nM and 200 nM 1:2.5 NLCplexes, respectively, in contrast to 80.2 ± 6.9 nM and 182.1 ± 27.9 nM for 100 nM and 200 nM 1:2.5 albuplexes. Nevertheless, the total amount of internalized 1:2.5 albuplexes was approximately 50 % higher than that of the corresponding NLCplexes. Interestingly, the uptake plateaued at a concentration of 100 nM, suggesting possible saturation of cell surface receptors or membrane binding sites at this dose.

In contrast, the uptake of 1:5 albuplexes occurred within the same time frame as that of the corresponding 1:5 NLCplexes (Fig. 7b,d). In 30 min, almost 50 % of 100 nM, and 47 % of 200 nM 1:5 NLCplexes were taken up. In comparison, approximately 21 % (21.2 ± 0.9 nM) of 100 nM and 66.8 % (133.6 ± 15 nM) of 200 nM 1:5 albuplexes were taken up. The data indicated that the uptake of 100 nM albuplexes was hindered. However, at a concentration of 200 nM, a markedly enhanced uptake rate was observed for the albuplexes indicating that the HSA coat significantly improved their internalization. Notably, the previously observed uptake plateau was surpassed in this case: internalization of 200 nM albuplexes was approximately 50 % higher than that of the corresponding NLCplexes, reaching 150 nM out of the 200 nM applied. Concerning the rate of uptake, the calculated rate constants were as follows: 98.7 ± 17 nM and 95.2 ± 15 nM for 100 and 200 nM NLCplexes, and 42.4 ± 4 nM and 267.4 ± 19 nM for 100 and 200 nM albuplexes (Fig. 7f). The greater impact of HSA on the 200 nM 1:5 formulation, compared to 1:2.5, may be attributed to the slightly lower surface charge of the 1:5 albuplexes which might decrease electrostatic repulsion by the extracellular matrix and the cell membrane, thereby facilitating faster and more efficient uptake (Öztürk et al., 2024).

In spite of the observed differences between 1:2.5 and 1:5 albuplexes in terms of uptake kinetics and quantity of taken up particles, the obtained data indicate that the HSA coat improved their cellular uptake. As a transport protein, HSA interacts with multiple proteins on the cellular surface (such as gp60, gp30, and gp18), and drives the uptake of the albuplexes to receptor-mediated endocytosis, circumventing their uptake through non-specific interactions with the cellular membrane (Larsen et al., 2016). A trait of receptor-mediated endocytosis is the need of a certain cargo threshold on the cell surface for uptake initiation, together with uptake saturation after a certain time point (Conner and Schmid, 2003; Kaksonen and Roux, 2018). Therefore, it is not surprising that in both cases (ratios 1:2.5 and 1:5) we observed a better effect at a concentration of 200 nM – a finding which suggests that the uptake is concentration-dependent. Furthermore, the uptake saturation occurred at 100 nM for the 1:2.5 formulation, and at 150 nM for the 1:5 formulation. The reason behind this might be the surface chemistry and the distribution of albumin on the surface of the nanoparticles. NLCplexes at a ratio of 1:5 demonstrate a higher surface charge allowing for a higher amount of HSA to be adsorbed on their surface and higher ligand coverage (Tucak-Smajić et al., 2023). Furthermore, the HSA on the surface of 1:5 albuplexes might be more loosely packed compared to 1:2.5 albuplexes. Indeed, literature suggests that the ligand surface density, organization, and presentation are essential for effective and efficient endocytosis, meaning that this might be one of the reasons for the better uptake of 1:5 albuplexes (Lee and Odom, 2015; Schubertová et al., 2015). What is more, at both ratios, and at both concentrations applied, the uptake of albuplexes is characterized by a zig-zag pattern, composed of several minimums and maximums. The reason behind this observation might be due to receptor saturation and receptor recycling (Manzanares and Ceña, 2020). Since there is a finite number of membrane receptors available to interact with ligands, after some time, all of them will be saturated. The receptors which are bound to cargo will undergo endocytosis, and once they are free, they will be recycled back to the membrane to interact with the available ligands (Disanza et al., 2009).

### 3.7 Metabolic inhibition and HSA competition studies

**Fig. 8:**
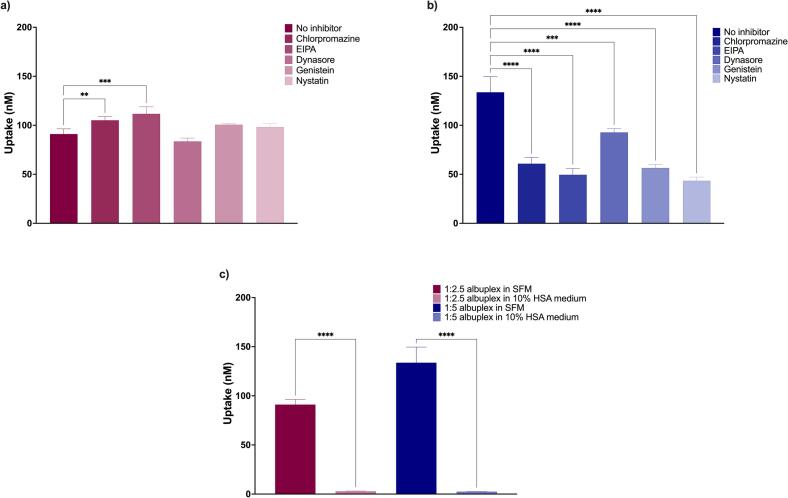
Metabolic inhibition studies of 1:2.5 (a) and 1:5 (b) albuplexes. Here, the uptake mechanism was tested by using various inhibitors of endocytosis. Furthermore, results obtained by studies done using free HSA as a competitive binder to the HSA cell surface receptors are presented in c. Data were analyzed using one-way ANOVA with *p*-values of 0.05 or less considered statistically significant (*p* < 0.05 (*), *p* < 0.01 (**), *p* < 0.001 (***), and *p* < 0.0001 (****).

To elucidate the mechanisms governing the uptake of albuplexes, metabolic inhibition studies were performed using a range of endocytosis inhibitors. Our recently published findings on the uptake of 1:2.5 and 1:5 NLCplexes indicated the involvement of multiple endocytic pathways, with clathrin-mediated endocytosis (CME) and macropinocytosis being the predominant routes (Ruseska et al., 2025). Nevertheless, the influence of HSA on the uptake mechanism of the 1:2.5 formulation remained inconclusive. As shown in Fig. 8a, treatment with metabolic inhibitors did not significantly alter uptake. Interestingly, a slight increase in internalization was observed following treatment with chlorpromazine and EIPA. The measured uptake in these cases was 105.1 ± 3 nM and 111.6 ± 7 nM, respectively, compared to 91 ± 5 nM in the absence of an inhibitor. These finding potentially suggests that inhibition of clathrin-mediated endocytosis or macropinocytosis triggers compensatory uptake via alternative pathways. In contrast, dynasore treatment resulted in a modest uptake reduction (83.5 ± 3 nM), implying partial dependence on a dynamin-mediated process.

For the 1:5 albuplexes, inhibitor treatment had a more pronounced effect (Fig. 8b). The uptake was reduced in the presence of all tested inhibitors, with EIPA and nystatin exhibiting the most substantial inhibitory effect, followed by chlorpromazine. The measured uptake in the presence of EIPA was 49.5 ± 6 nM, in the presence of nystatin it was 43.4 ± 3 nM, and in the presence of chlorpromazine we obtained 60.8 ± 6 nM. In contrast, the uptake in the absence of inhibitors was 133.6 ± 15 nM. There results suggest that the internalization of 1:5 albuplexes predominantly occurs through clathrin-mediated endocytosis and macropinocytosis. However, given that genistein also influenced the uptake, caveolae-mediated endocytosis (CvME) cannot be excluded.

Metabolic inhibition data indicate that the uptake of 1:5 albuplexes is a complex interplay of multiple mechanisms (CME, CvME and macropinocytosis). In contrast, no solid conclusion can be made concerning the uptake of 1:2.5 albuplexes. The activation of multiple uptake pathways by 1:5 albuplexes might be the reason behind their faster and higher uptake reported earlier in the text. Literature suggests that fibroblasts, such as the 3T3-L1 cells used in our study, lack the gp60 receptor, and take up albumin via CME through the gp30 and gp18 receptors, which represent scavenger receptors (Ishima et al., 2022; Ji et al., 2024). Furthermore, non-specific uptake through macropinocytosis has also been reported for albumin (Miao et al., 2020; Sutton et al., 2022). Based on this, it is likely that 1:5 albuplexes are taken up by 3T3-L1 cells through CME and macropinocytosis. Further studies are needed in order to evaluate the receptor involved in the uptake of this formulation, as well as to analyze in depth the mechanism of uptake of 1:2.5 albuplexes.

To validate our hypothesis that HSA is the primary driver of the albuplex uptake, we performed uptake studies using cells that were pre-treated with 10 % HSA (as a medium supplement). As illustrated in Fig. 8c, pre-incubation with HSA reduced the uptake of both 1:2.5 and 1:5 albuplexes compared to their uptake in serum-free medium (SFM). The obtained data demonstrated that only 3 nM of 1:2.5 albuplexes and 2.5 nM of 1:5 albuplexes were internalized in HSA-supplemented serum. In contrast, 91.06 ± 5 nM of 1:2.5 albuplexes and 133.6 ± 15 nM of 1:5 albuplexes were taken up in serum-free medium. These results suggest a potential competitive interaction between free HSA in the medium and the albumin adsorbed on the surface of albuplexes for binding to the cell surface receptors. This is in accordance with previously published literature, where free albumin in the medium hindered the uptake of albumin-coated nanoparticles (Mo et al., 2007).

### 3.8 Confocal laser scanning microscopy (CLSM)

**Fig. 9:**
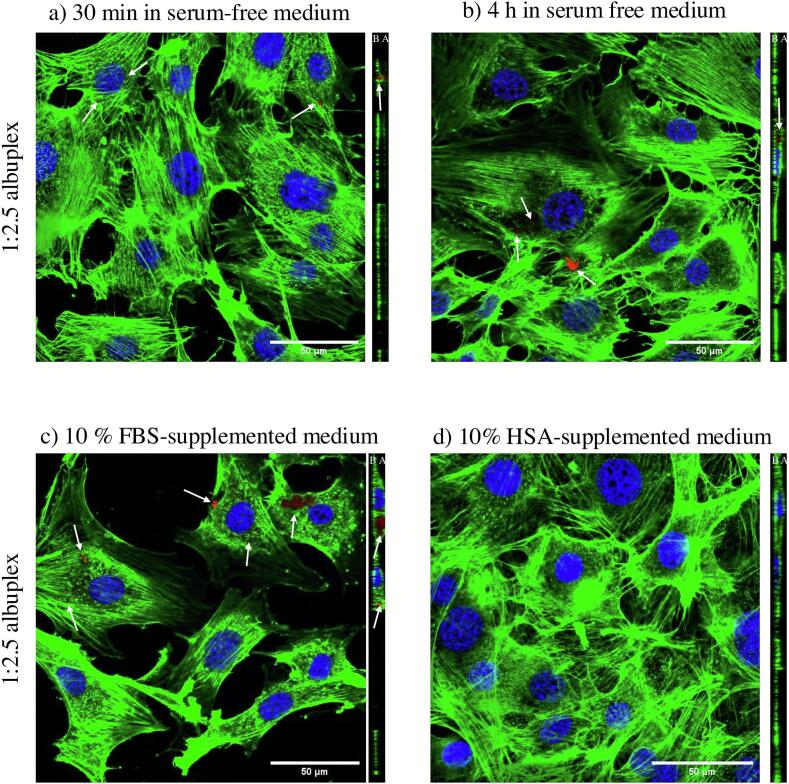
CLSM images of 3T3-L1 cells transfected using 1:2.5 albuplexes for a) 30 min and b) 4 h in serum-free medium. Furthermore, cells were transfected in serum conditions (c), as well as in the presence of 10 % free HSA (d). The complexes were labelled using Cy-3 tagged non-targeting miRNA control (FluoNTC) and are marked with a white arrow. The actin cytoskeleton was counterstained using Alexa Fluor™ 488 Phalloidin (green), whereas the nuclei were stained using DAPI (blue). The obtained z-stacks are marked with A and B for apical and basal side of the cells, respectively. (For interpretation of the references to colour in this figure legend, the reader is referred to the web version of this article.)

**Fig. 10:**
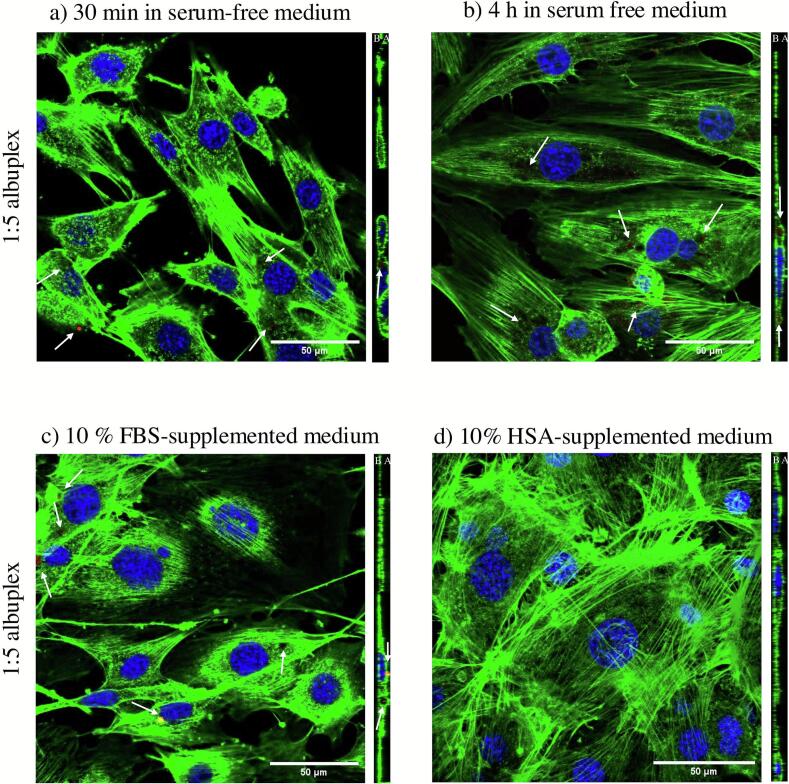
CLSM images of 3T3-L1 cells transfected using 1:5 albuplexes for a) 30 min and b) 4 h in serum-free medium. Furthermore, cells were transfected in serum conditions (c), as well as in the presence of 10 % free HSA (d). The complexes were labelled using Cy-3 tagged non-targeting miRNA control (FluoNTC) and are marked with a white arrow. The actin cytoskeleton was counterstained using Alexa Fluor™ 488 Phalloidin (green), whereas the nuclei were stained using DAPI (blue). The obtained z-stacks are marked with A and B for apical and basal side of the cells, respectively. (For interpretation of the references to colour in this figure legend, the reader is referred to the web version of this article.)

To further validate the uptake data, we utilized confocal laser scanning microscopy to visualize the albuplexes and assess their intracellular localization. For this purpose, the albuplexes were labelled using a Cy-3 tagged non-targeting miRNA control (FluoNTC), visible as red. The actin cytoskeleton was stained using Alexa Fluor™ 488 Phalloidin (green), whereas the nuclei were stained using DAPI (blue). Both 1:2.5 and 1:5 albuplexes were clearly visible within cells after 30 min of incubation. As shown in Fig. 9, Fig. 10, the albuplexes appeared as small red vesicles associated with the actin cytoskeleton (as seen in the orthogonal projection). After 4 h of incubation, the number of red vesicles increased, particularly for the 1:5 complexes (Fig. 10b), further supporting the semi-quantitative data previously presented. The punctate distribution of the albuplexes inside the cells is in accordance with previously published data (Galisteo-González et al., 2018).

We also assessed the uptake of 1:2.5 (Fig. 9c) and 1:5 (Fig. 10c) albuplexes in the presence of serum proteins by incubating the albuplexes in medium supplemented with 10 % FBS prior to transfection The confocal images indicate that serum proteins did not impact the uptake; both formulations showed similar internalization profiles, which may be attributed to the enhanced stability of albuplexes in serum compared to uncoated NLCplexes. Our findings are in accordance with published literature, which suggests that using HSA as a dysopsonin can lead to increased cellular uptake in the presence of serum proteins, together with improving the stability of the nanoparticles (Bozzer et al., 2024; Singh et al., 2021).

In contrast, pre-incubation with medium supplemented with 10 % HSA completely inhibited the uptake of albuplexes, confirming the findings from the previously presented semi-quantitative analysis (Fig. 9c and 10c). The reduced uptake of albuplexes in the presence of free HSA – but not free BSA – is likely due to structural differences between the two proteins and their distinct receptor recognition profiles. Additionally, the dynamic nature of the protein corona may allow free HSA to displace or mask functional HSA on the surface of albuplexes, thereby interfering with receptor-mediated uptake. The result reinforces the hypothesis that the albumin coat on albuplexes is the primary driving force behind their uptake.

### 3.9 In vitro efficacy studies in 3T3-L1 preadipocytes

Recent research has revealed the importance of miRNA in adipocyte development, metabolic function, proliferation, and differentiation (Heyn et al., 2020). Among them, miR-27a has been identified to have anti-adipogenic properties as it inhibits the process of adipocyte differentiation by suppressing PPARγ expression (Kim et al., 2010). In addition, a decrease in miRNA-27a levels is thought to be related to adipose tissue dysfunction in obesity (Cruz et al., 2017). In our previous study, we demonstrated the efficacy of miR-27a/cNLC complexes in preventing the differentiation of 3T3-L1 preadipocytes into mature adipocytes. We also confirmed that our developed cNLC formulation serves as a versatile carrier for different miRNA types (miRNA-27a, miRNA-NTC, miRNA-FluoNTC), producing NLCplexes with similar physicochemical properties in terms of particle size and surface charge (Tucak-Smajić et al., 2023).

**Fig. 11:**
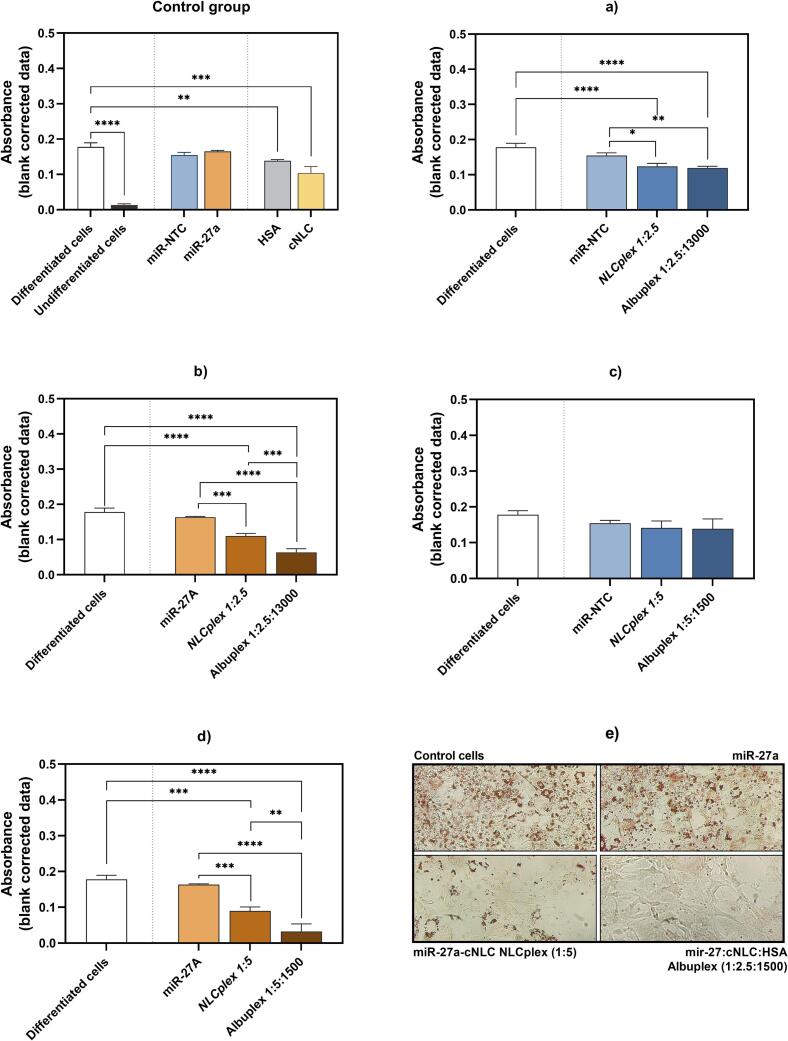
Absorbance of accumulated ORO dye in lipid droplets on day 6 of 3T3-L1 cell differentiation. The study evaluates how complexation with cNLC affects differentiation compared to free miRNA, as well as the impact of HSA coating on NLCplexes. The control group includes differentiated and undifferentiated cells, free NTC, free miRNA-27a, free HSA, and cargo-free cNLC. The experimental groups are as follows: (a) miRNA-27a, miRNA-27a:cNLC NLCplexes (1:2.5), and miRNA-27a:cNLC:HSA albuplexes (1:2.5:1300), (b) miRNA-NTC, miRNA-NTC:cNLC NLCplexes (1:2.5), and miRNA-NTC:cNLC:HSA albuplexes (1:2.5:1300), (c) miRNA-27a, miRNA-27a:cNLC NLCplexes (1:5), and miRNA-27a:cNLC:HSA albuplexes (1:5:1500), (d) miRNA-NTC, miRNA-NTC:cNLC NLCplexes (1:5), and miRNA-NTC:cNLC:HSA albuplexes (1:5:1500). (e) Light microscopy images of differentiated 3T3-L1 cells on day 6 after ORO staining, including control cells, free miRNA-27a, miRNA-27a:cNLC NLCplexes (1:5), and miRNA-27a:cNLC:HSA albuplexes (1:5:1500). Results are presented as mean ± SD (*n* = 6). Statistical significance: *p* < 0.05 (*), p < 0.01 (**), p < 0.001 (***), and *p* < 0.0001 (****).

In the present study, we investigated whether decorating NLCplexes with HSA could enhance the proven antiadipogenic effect of miR-27a/cNLC complexes (Fig. 11). Since the highest efficacy of NLCplexes was observed at a miRNA-27a concentration of 100 nM, albuplexes were prepared at mass ratios of 1:2.5:1300 and 1:5:1500 to contain a final miRNA concentration of 100 nM (miRNA-27a or miRNA-NTC). The staining intensities of ORO dye accumulated within the lipid droplets in cells were determined by absorbance measurements (Fig. 11. a-d), while light microscopy was used to assess the effects of free miRNA, NLCplexes, and albuplexes on cell morphology following differentiation (Fig. 11e).

The results confirmed that neither free miR-27a nor NTC reduced lipid droplet formation (Fig. 11), which is expected due to the low cellular uptake. However, cargo-free cNLC and HSA significantly influenced the accumulation of ORO dye in cells, compared to differentiated cells (*p* < 0.001, and *p* < 0.01, respectively). The incorporation of OA into the cNLC formulation and achieving the cationic surface charge, not only promotes the stability of the formulation but allows easier interaction of carriers with the cell membrane, leading to the internalization of the nanoparticles and consequently causing stress to the cells that impair the differentiation process. Although HSA is not known for having anti-adipogenic properties, the differentiation process of preadipocytes into mature adipocytes is affected following the internalization of free HSA by cells.

Although free miR-27a did not influence the differentiation of cells, its incorporation in miR-27a NLCplexes at both mass ratios of 1:2.5 and 1:5 significantly reduced the ORO accumulation in cells compared to differentiated cells and free miRNA (Fig. 11a,c), indicating that miR-27a/cNLC complexes can serve as an effective DDS for miR-27a, which is aligned with our previous study (Tucak-Smajić et al., 2023). However, the decorating of miR-27a/cNLC complexes with HSA at mass ratios 1:2.5:1300 and 1:5:1500 further enhanced the anti-adipogenic properties of these NLCplexes. As shown in Fig. 11b and d the albuplexes significantly decreased the intensities of ORO accumulated in cells compared to control cells, NLCplexes, and free miR-27a. Among them, the albuplex at a mass ratio of 1:5:1500 was more effective and decreased the absorbance to 0.032 ± 0.021 compared to 0.063 ± 0.011 (1:2.5:1300) and 0.178 ± 0.012 (control cells).

On the other hand, NTC/cNLC complexes (1:5) and albuplexes (1:5:1500) did not affect the differentiation process or reduce the formation of lipid droplets (Fig. 11d), while for complexes at a mass ratio of 1:2.5 and albuplex at a mass ratio 1:2.5:1300 a reduction in ORO accumulation is noticed (Fig. 11b). Given that these results cannot be attributed to the antiadipogenic properties of miRNA, it seems that free cNLC or HSA in the formulation can also influence the differentiation process to some extent.

The microscopic analysis confirmed the semi-quantitative results and showed that 3T3-L1 cells treated with miR-27a:cNLC complexes (1:5) and albuplexes (miR-27a:cNLC:HSA mass ratio 1:5:1500) exhibited significantly lower lipid accumulation than cells treated with free miR-27 (Fig. 11e). In addition, morphological changes were observed, with NLCplex*-* and albuplex-treated cells being less round and maintaining their fibroblast-like appearance, compared to the larger, lipid-laden morphology of fully differentiated adipocytes in the control group and cells treated with miR-27a. Since miR-27a is a negative regulator of PPARγ, these morphological changes are likely associated with changes in the expression of this adipogenic marker, which is associated with reduced differentiation of the cells.

### 3.10 Future directions

Although NLCplexes containing miR-27a showed great potential for use as DDS for treating or preventing obesity and obesity-related diseases, our findings demonstrate that HSA precoating of NLCplexes further enhances the antiadipogenic potential of these complexes and may serve as a promising strategy to improve miRNA-based therapeutics. Therefore, in our future research, we aim to evaluate the potential of HSA-coated nanoparticles as a targeted delivery platform for adipose tissue by conducting in vivo studies with local administration of albuplexes in mouse models. Given the currently limited evidence linking HSA coating to improved adipose tissue targeting, these investigations will help address this gap by monitoring miRNA accumulation, internalization, and biodistribution within adipose tissue. We hypothesize that HSA decoration could facilitate nanoparticle extravasation from local blood vessels through interactions with endothelial gp60 receptors and promote uptake into adipocytes, as reported in other tissues, such as endothelial and tumor tissues (Hyun et al., 2018; Shahapurmath et al., 2025; Taguchi et al., 2021).

## 4 Conclusion

The presented work demonstrates that cationic NLCplexes (complexes between cationic NLCs and negatively charged miRNA) can be successfully coated with HSA to obtain the so-called albuplexes. NLCplexes at two different ratios (1:2.5 and 1:5) were used for coating purposes, and both demonstrated desirable physicochemical properties after HSA coating, together with favourable biocompatibility. Together with DLS, ELS, and AFM, the albuplexes were characterized using CD and UV/Vis spectroscopy, in order to confirm the stability of HSA on the surface of the nanoparticles. By adsorbing on the surface of NLCplexes the structure of HSA was not only preserved, but also enhanced, as demonstrated by increased a-helicity at various temperatures. Furthermore, using HSA as a dysopsonin led to higher stability of NLCplexes in serum-supplemented medium, with 1:5 albuplexes demonstrating superior stability compared to 1:2.5 albuplexes.

Cellular uptake studies demonstrated that albuplexes were taken up successfully in 3T3-L1 cells, with the 1:5 formulation demonstrating higher and faster uptake compared to the 1:2.5 formulation. The reasoning behind this might be the complex network of endocytic mechanisms involved in its uptake, as demonstrated by the metabolic inhibition studies, suggesting that CME and macropinocytosis are involved. Also, this formulation showed a lower surface charge in serum-free medium, which might lead to faster uptake due to lower electrostatic repulsion by the ECM and cell membrane. On the contrary, inhibition studies turned out to be inconclusive for 1:2.5 albuplexes, leaving an open question and possibilities for future in-depth investigations. The uptake of this formulation was slower compared to the respective uncoated NLCplexes, probably due to electrostatic repulsion by the ECM. The uptake of both formulations was further confirmed using CLSM.

Uptake studies performed in the presence of serum proteins demonstrated that the internalization of 1:2.5 and 1:5 albuplexes was not hindered, likely due to the HSA coat on the surface. Furthermore, when transfection was performed in in presence of free HSA, the uptake of albuplexes was diminished, suggesting that the interaction between HSA and a cell surface receptor is the driving force behind their internalization.

Our findings were further supported by efficacy studies done in 3T3-L1 cell, where albuplexes showed superior reduction of lipid droplet formation after successful release of miRNA-27a compared to NLCplexes and miRNA-27a alone.

Nanostructured lipid carriers are attractive candidates for the delivery of numerous active compounds, which offer versatility in terms of method of preparation and ways of administration. As we have demonstrated, their surface properties can be successfully modified to achieve higher stability, more specific targeting, efficient cellular uptake, and higher efficacy.

## CRediT authorship contribution statement

**Ivana Ruseska:** Writing – original draft, Investigation. **Amina Tucak-Smajić:** Writing – original draft, Investigation. **Ivan Vidaković:** Methodology, Investigation. **Karin Kornmüller:** Methodology, Investigation. **Edina Vranić:** Supervision. **Andreas Zimmer:** Supervision, Project administration.

## Declaration of competing interest

None of the authors have a conflict of interest regarding the present study or have anything to disclose.

## Data Availability

Data will be made available on request.

## Appendix A Supplementary data

Supplementary material Image 1
