# Supplementary material for: Optimizing microRNA delivery via albumin-decorated nanostructured lipid carriers
Source: Int J Pharm X. 2025 Nov 7;10:100441. doi: 10.1016/j.ijpx.2025.100441 (PMC12663038; doi:10.1016/j.ijpx.2025.100441)
Supplement: Supplementary file 1 — Supplementary material [file mmc1.docx]

**Supplementary Material**

**S1. Influence of the incubation time on the preparation of albuplexes**

The efficiency of pharmaceutical and nanotechnological systems may be affected by various variables such as incubation time and temperature, which makes developing non-viral gene delivery systems a challenging task. Currently, no standard guidelines regarding the optimal incubation time are provided, as these depend on the specific carrier system used. For example, (Wang et al., 2021), reported an incubation period of 15 minutes for albumin-coated siRNA-liposome complexes, whereas in the study of (Kummitha et al., 2012) EHCO/siRNA nanoparticles were incubated for 30 minutes with bovine serum albumin. However, to the best of our knowledge, no studies described the coating of miRNA:NLC complexes with albumin.

The influence of incubation times ranging from 5 to 60 minutes on the physicochemical properties of the albuplexes at miRNA:OA:HSA mass ratios of 1:2.5:1300 and 1:5:1500 is presented in Table S1.

**Table S1.** Effect of incubation time on the physicochemical properties of *albuplexes*

| *Incubation time* | ***miRNA:OA:HSA mass ratio*** | | | | | |
| --- | --- | --- | --- | --- | --- | --- |
|  | ***1:2.5:1300*** | | | ***1:5:1500*** | | |
|  | *z-ave (nm)* | *PdI* | *ZP (mV)* | *z-ave (nm)* | *PdI* | *ZP (mV)* |
| *5 min* | 137.6 ± 2.8 | 0.173 ± 0.004 | −21.8 ± 0.8 | 135.8 ± 2.3 | 0.175 ± 0.005 | −18.2 ± 0.3 |
| *15 min* | 142.7 ± 2.4 | 0.165 ± 0.014 | −20.5 ± 1.4 | 139.1 ± 0.9 | 0.175 ± 0.005 | −19.3 ± 1.1 |
| *30 min* | 141.7 ± 1.1 | 0.171 ± 0.007 | −20.4 ± 0.6 | 139.6 ± 2.0 | 0.183 ± 0.008 | −18.9 ± 0.8 |
| *60 min* | 145.8 ± 0.8 | 0.170 ± 0.006 | −20.8 ± 0.6 | 143.9 ± 3.3 | 0.179 ± 0.008 | −18.6 ± 0.5 |

The results in Table S1 indicate that incubation time has no significant effect on the physicochemical properties of formed albuplexes (p > 0.05). As the incubation time increased, there was a slight increase in particle size for both miRNA:OA:HSA mass ratios, but the PdI remained stable and below 0.2, indicating narrow size distributions and consistent formulations. Additionally, a slight decrease in ZP values was observed over time (p > 0.05). These findings demonstrate that incubation time has minimal impact on the stability and surface properties of the albuplexes, with effective decoration of the complexes over time. Therefore, for further studies, albuplexes were prepared with a 5-minute incubation period, which minimizes preparation time while maintaining the desired physicochemical properties of albuplexes.

**S2. Stability of albuplexes in different dilution media**

Complex formation between miRNAs, cNLCs, and HSA is a thermodynamically spontaneous process that represents a modification of the surface of positively charged NLCplexes with negatively charged albumin, resulting in electrostatic interactions at pH values higher than the isoelectric point of HSA. Therefore, environmental factors, including salt concentration, can influence these interactions. The effect of different dilution media: RNase-free water, Zeta water, PBS, and low glucose serum-free DMEM on the physicochemical properties of albuplexes at mass ratios of miRNA:OA:HSA of 1:2.5:1300 and 1:5:1500, are shown in Figure S2.

******

**Figure S2.** Physicochemical properties of albuplexes at mass ratios of 1:2.5:1300 and 1:5:1500 (miRNA concentration: 100 nM) in different dilution media, represented by particle size (a, b) and zeta potential (c, d). Data were analyzed using one-way ANOVA, with p-values of 0.05 or less considered statistically significant (*p < 0.05; **p < 0.01; ***p < 0.001).

The results shown in Figure S2 illustrate the variations in the physicochemical properties of albuplexes in different dilution media. At both mass ratios (1:2.5:1300 and 1:5:1500), changes in particle size were observed, indicating that the composition of the medium plays a crucial role in the stability of the complexes. The albuplexes were mainly prepared in RNase-free water to minimize the risk of nucleic acid degradation, and thus they are serving as a control to assess the impact of other media.

Zeta water, which is commonly used as dilution medium for the measurement of ZP, is a distilled water with adjusted conductivity containing a defined NaCl ion concentration of 0.5 mM. When albuplexes were diluted in this media, only slight changes in particle size were observed compared to those measured in RNase-free water, indicating that low ionic strength minimally affects particle aggregation. On the other hand, the measured ZP values were lower than those measured in RNase-free water, due to the presence of NaCl, which can compress the electrical double layer and consequently reduce surface charge (Figure S2c,d).

In contrast, PBS and serum-free low glucose DMEM induced noticeable changes, particularly in the particle size of albuplexes at mass ratio 1:5:1500. This effect may be attributed to ionic interactions and potential aggregation effects for albuplexes prepared in PBS. The low-glucose DMEM contains amino acids, vitamins, and inorganic salts, which could potentially influence the particle size and stability of the complexes. In this case, particle size of albuplexes was significantly reduced compared to other media, accompanied by slightly higher ZP values above –10 mV.

PBS, an isotonic buffer commonly used in cell culture studies, closely mimics physiological pH, osmolarity, and ion concentrations. Its composition (137 mM NaCl, 2.7 mM KCl, 8 mM Na_2_HPO_4_, and 2 mM KH_2_PO_4_) results in high ionic strength, which can compress the diffuse layer and reduce ZP values, similar to those measured in low-glucose DMEM, as shown in Figure S2c,d. Additionally, the adsorption of molecules from the media may lead to changes in particle size and surface properties (Bhattacharjee, 2016).

**S3.** Surface morphology of cNLCs, NLCPlexes, and albuplexes

The AFM method was used to investigate the surface properties of the cargo-free NLC formulation, the NLCplexes (1:2.5, w/w), and the albuplexes (1:2.5:1300, w/w/w). The height images are shown in Figure S3.

| 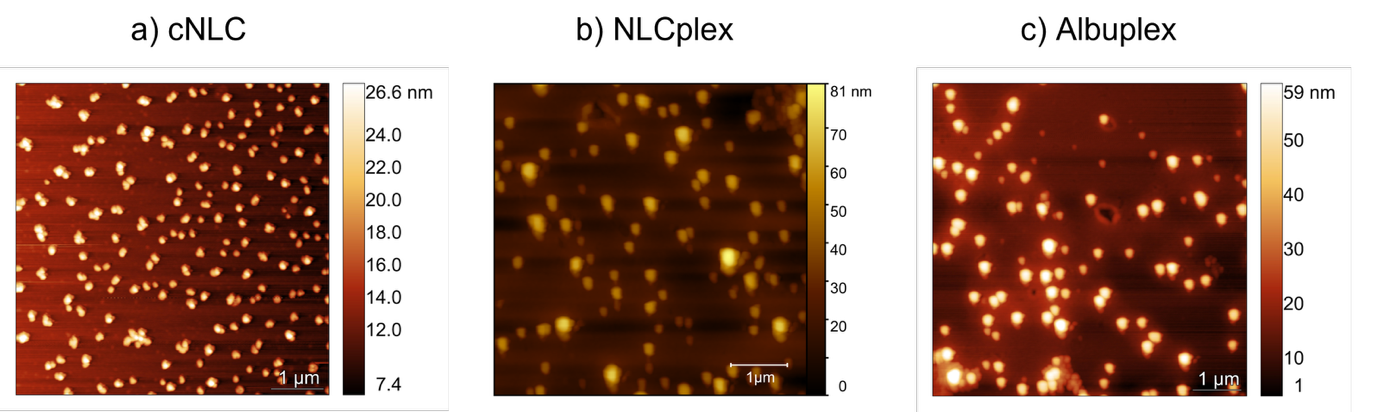 |
| --- |
| **Figure S3.** Atomic force microscope images of the cNLC formulation, the NLCplexes (1:2.5, w/w), and the *albuplexes* (1:2.5:1300, w/w). |

The AFM images show slight variations between the cargo-free cNLCs and the complexes with miRNA in particle height and surface uniformity, which can be attributed to electrostatic complexation (Figure S3). The additional changes in the morphology of the albuplexes, including a smoother and more uniform surface, are shown in Figure S3c. The observed AFM data suggest that both miRNA complexation and albumin coating play a crucial role in modulating the physicochemical properties of the nanoparticles. While miRNA binding alters the native cNLC structure, albumin coating appears to have a stabilizing effect that can improve the performance of the nanoparticles in physiological environments.


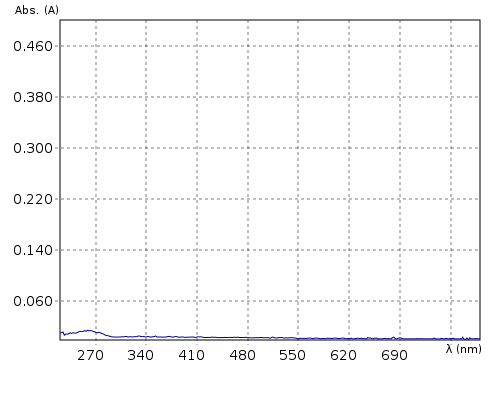


miRNA absorbance

**Figure S4.** UV/Vis spectra for naked miRNA


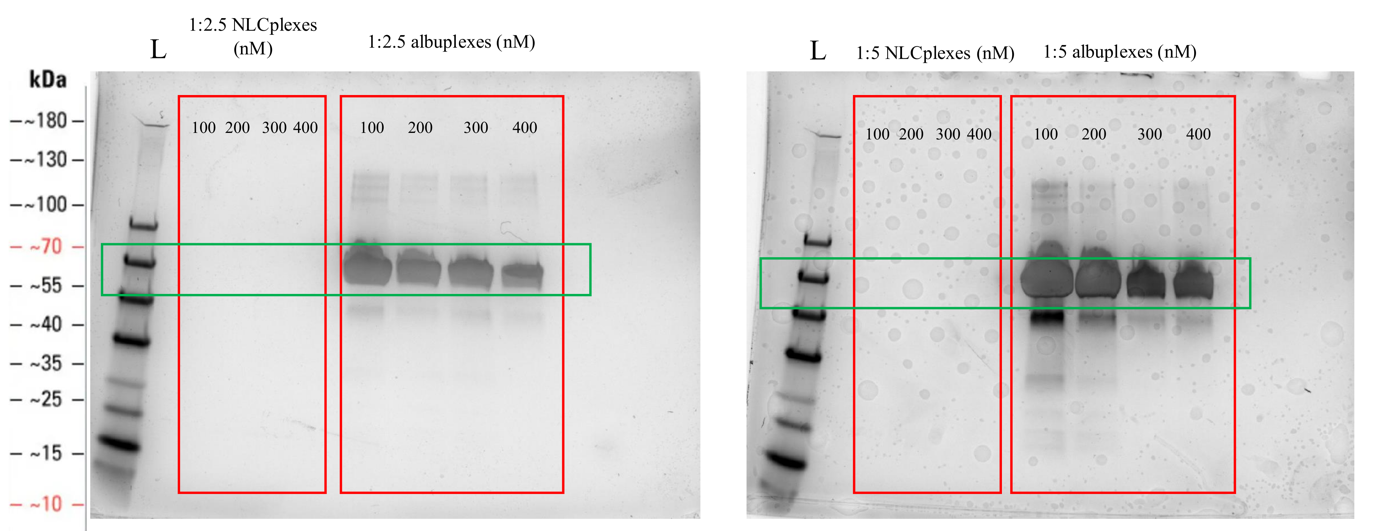


**Figure S5**. Qualitative control of the HSA coating of 1:2.5 and 1:5 NLCplexes and the formation of albuplexes using SDS-PAGE. The complexes were produced in MQ water.


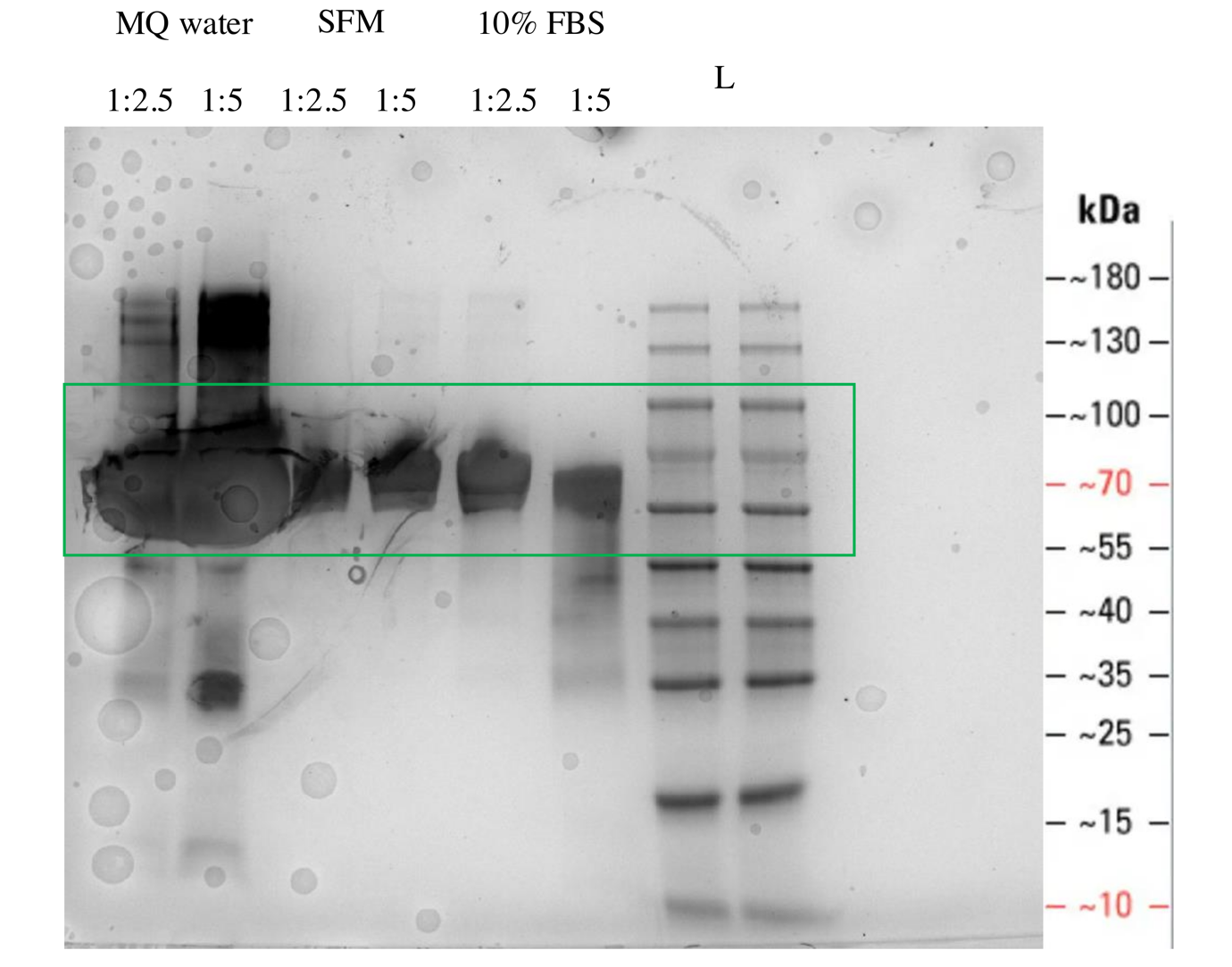


**Figure S6**. Qualitative analysis of the protein corona formed on 1:2.5 and 1:5 complexes using gel electrophoresis. The complexes were incubated in either MQ water, serum-free medium, or medium containing 10%FBS, and water. The obtained data indicate that the most abundant protein attached on their surface is HSA. The darker bands observed when complexes were incubated in MQ water are due to overexpose of the gel during development.

**References**

Bhattacharjee, S., 2016. DLS and zeta potential - What they are and what they are not? Journal of Controlled Release 235, 337–351. https://doi.org/10.1016/j.jconrel.2016.06.017

Kummitha, C.M., Malamas, A.S., Lu, Z.R., 2012. Albumin pre-coating enhances intracellular siRNA delivery of multifunctional amphiphile/siRNA nanoparticles. Int J Nanomedicine 7, 5205–5214. https://doi.org/10.2147/IJN.S34288

Wang, D., Li, H., Chen, W., Yang, H., Liu, Y., You, B., Zhang, X., 2021. Efficient tumor-targeting delivery of siRNA via folate-receptor mediated biomimetic albumin nanoparticles enhanced by all-trans retinoic acid. Materials Science and Engineering C 119, 111583. https://doi.org/10.1016/j.msec.2020.111583
